# The Evolutionary Basis of Naturally Diverse Rice Leaves Anatomy

**DOI:** 10.1371/journal.pone.0164532

**Published:** 2016-10-28

**Authors:** Jolly Chatterjee, Jacqueline Dionora, Abigail Elmido-Mabilangan, Samart Wanchana, Vivek Thakur, Anindya Bandyopadhyay, Darshan S. Brar, William Paul Quick

**Affiliations:** 1 C4 Rice Center, Genetics and Biotechnology Division, International Rice Research Institute, Los Baños, DAPO BOX 7777, Metro Manila, Philippines; 2 Plant Breeding, Genetics and Biotechnology Division, International Rice Research Institute, Los Baños, DAPO BOX 7777, Metro Manila, Philippines; 3 Department of Animal and Plant Sciences, University of Sheffield, Sheffield S10 2TN, United Kingdom; National Institute of Plant Genome Research, INDIA

## Abstract

Rice contains genetically and ecologically diverse wild and cultivated species that show a wide variation in plant and leaf architecture. A systematic characterization of leaf anatomy is essential in understanding the dynamics behind such diversity. Therefore, leaf anatomies of 24 *Oryza* species spanning 11 genetically diverse rice genomes were studied in both lateral and longitudinal directions and possible evolutionary trends were examined. A significant inter-species variation in mesophyll cells, bundle sheath cells, and vein structure was observed, suggesting precise genetic control over these major rice leaf anatomical traits. Cellular dimensions, measured along three growth axes, were further combined proportionately to construct three-dimensional (3D) leaf anatomy models to compare the relative size and orientation of the major cell types present in a fully expanded leaf. A reconstruction of the ancestral leaf state revealed that the following are the major characteristics of recently evolved rice species: fewer veins, larger and laterally elongated mesophyll cells, with an increase in total mesophyll area and in bundle sheath cell number. A huge diversity in leaf anatomy within wild and domesticated rice species has been portrayed in this study, on an evolutionary context, predicting a two-pronged evolutionary pathway leading to the ‘*sativa* leaf type’ that we see today in domesticated species.

## Introduction

Rice leaf is composed of diverse cell types like, mesophyll cells (MC), bundle sheath cells (BSC), epidermal cells (EP), bulliform cells (BL), stone cells (ST), and vascular bundles (VB) with xylem and phloem and their associated companion cells. The equi-facial dorso-ventrally flattened rice leaf originates from the leaf primordial cells in the SAM or the shoot apical meristem [1]. Usually, changes in the cell division and cell expansion during axis formation, tissue differentiation, and tissue specification finally determine the leaf shape [2]. A synchronized activity of all these cellular modules effectively controls the leaf function [3]. Rice and its wild species possess huge diversity in plant and leaf phenotypes [4, 5]. This important crop species belongs to grass genus *Oryza* that are formed by a total of 24 different *Oryza* species. Overall, these species contain 11 diverse rice genomes from AA to KKLL, named differently according to their genetic distance [4–6]. The most recently evolved species in the history of rice are the cultivated rice species *Oryza sativa* and *Oryza glaberrima* that harbor the AA genome [7]. For the rest of the species, the level of genetic and reproductive diversity traditionally increases in an A to Z alphabetical order across the genomes.

Leaf structure strongly controls leaf photosynthesis [8, 9] and plays a key role in every step starting from light interception up to the biochemical fixation of carbon dioxide. Engineering the leaf structure of cultivated rice could, therefore, be of direct interest to current research efforts that aim to increase photosynthetic efficiency and thereby achieve improved yields [10–12]. Despite leaf anatomy being a central component that determines leaf photosynthesis and gas exchange, very little attention has been paid to quantify the diversity of leaf anatomical traits within *Oryza* to use for genetic improvement or plant breeding programs in rice. Unfortunately, the functional significance of leaf structure, especially at the cellular level, and its regulation is still not very clear in rice.

Until now, research on leaf structure in cultivated rice is mostly confined to leaf shape [13, 14] and leaf angle [15, 16]. There have been some studies to understand the function of bulliform cells [17–19], and more recently the variation in vein patterning and mesophyll architecture in a mutant population of IR64 [20]. Of late, there has been growing interest in the characterization of the foliar structure of wild rice ancestors [21, 22], which include some of the wild species used in this structure-function study. However, none of these studies have included all the *Oryza* species nor facilitated an evolutionary analysis as what this paper presents.

The genetics of the genus *Oryza* is the most extensively studied among the grasses [6, 23–25]. Therefore, a systematic genome-wide characterization of leaf structure in diverse rice species might give an idea of how the structure of rice leaves has evolved in nature and also unravel new traits that could be engineered into rice to increase its productivity. Indeed, here we have shown the extensive variation in leaf anatomical components in different rice species that contain different genetic makeup. Moreover, the leaf cellular diversity is compared in a 3-dimensional (3D) volume basis rather than comparing them just in a conventional 2D area basis, and we discuss this further with a phylogenetic context.

## Materials and Methods

### Plant materials

Twenty four *Oryza* species were characterized in terms of leaf morphology and anatomy. The grouping of these 24 species under different genomes are as follows: *Oryza sativa*, *O*. *glaberrima*, *O*. *nivara*, *O*. *barthii*, *O*. *meridionalis*, *O*. *rufipogon*, *O*. *longistaminata*, and *O*. *glumaepatula* belong to the most recent AA genome; *O*. *punctata* belongs to the BB genome; *O*. *minuta* belongs to the allo-tetraploid BBCC genome; *O*. *eichingeri*, *O*. *officinalis*, and *O*. *rhizomatis* belong to the CC genome; *O*. *alta*, *O*. *latifolia*, and *O*. *grandiglumis* belong to the allo-tetraploid CCDD genome; *O*. *australiensis* belongs to the EE genome; *O*. *brachyantha* belongs to the FF genome; *O*. *granulata* and *O*. *meyeriana* belong to the GG genome; *O*. *ridleyi*, and *O*. *longiglumis* belong to the allo-tetraploid HHJJ genome; *O*. *schlechteri* belongs to the allo-tetraploid HHKK genome; and finally *O*. *coarcatata* belongs to the allo-tetraploid KKLL genome (4, 5, 6). Conventionally, the genomes are genetically as distant from the AA genome as their alphabetical order suggests.

All the *Oryza* species were grown in a screen-house at the International Rice Research Institute, Philippines (14.1667° N, and 121.2167° E). Fully expanded leaves from 10 different clonally propagated plants of each accession were characterized at the vegetative stage.

### Sampling and leaf morphology characterization

Leaf types were determined based on both leaf blade length (LL, measured from base to tip of the fully expanded leaf blade) and width (LW, measured from margin to margin at the middle portion of the leaf blade). Samples for vein counting and leaf anatomy imaging were taken from the middle portion of the leaves. The leaves were fixed in FAA (50% ethanol, 5% (v/v) acetic acid and 3.7% (v/v) formaldehyde) for further histological studies.

### Counting of veins

Rice veins appear as white parallel bands on a green background on the leaf surface. Veins were imaged using a bright field microscope (Olympus BX51) at both the left and right sides of the midrib and at different locations lengthwise along the leaf. Vein number per mm was counted using Image J software (Wayne Rasband, National Institute of Health, USA).

### Leaf anatomical study

For detailed leaf anatomical studies, several sections (at least 3) were viewed and scored. For uniformity, only the middle part of a leaf section, taken from middle of the leaf, was used for analysis and scoring of different anatomical characters. Detailed anatomical information was obtained from leaf transverse (TS), longitudinal (LS), and paradermal sections (PS) of either free hand (50 μm thick) or microtome cut thin sections (20 μm). Images of these sections were taken using the Olympus BX51 compound microscope and photographed with the attached Olympus DP71 digital image documentation system. For thin sections, fixed leaves were dehydrated in a series of graded ethanol and embedded in Spurr’s resin with further graded infiltration series and were sectioned using an ultra-microtome (MT2-B, DuPont-Instruments-Sorvall, Newtown, CT, USA), followed by staining in 0.05% Toluidene Blue [26]. A Spinning Disc Fluorescent microscope (Olympus BX52) was used to collect fluorescence images showing cell boundaries. Anatomical parameters were quantified using Image J software.

### Defining leaf anatomical traits

**Fig 1:**
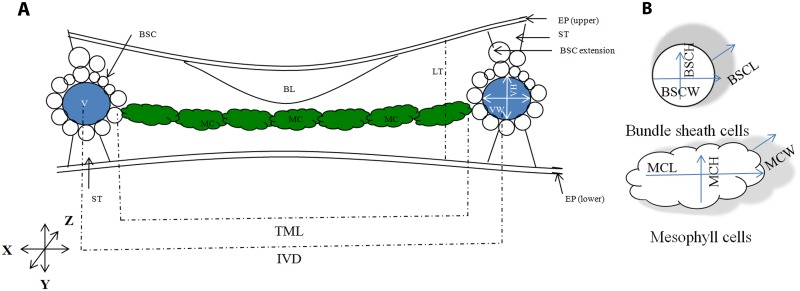
A schematic representation of a rice leaf shows different cell types and three different leaf dissection axes; used to calculate cell size, number, and volume. For ease of viewing, bulliform and epidermal cells are not shown in detail. (A) Rice leaf transverse section. The position of major leaf cells: mesophyll cell (MC, coloured green), bundle sheath cells (BSC, coloured white), vein (V, coloured blue), bulliform cells (BL), stone cells (ST), and epidermal layers. In rice, inter-veinal distance is filled by a number of elliptic mesophyll cells. Veins are surrounded by a wreath of bundle sheath cells and crowned by bundle sheath cell extension above the main circle. Large bulliform cells are present only at the adaxial side of the leaf. Stone cells are present at both the abaxial and adaxial end of the vein. X, Y and Z represent the three growth axes where, X represents the leaf lateral axis, Y represents the leaf abaxial-adaxial axis, and Z represents the leaf longitudinal or the proximo-distal axis. The long axis of the mesophyll cell is perpendicular to the vein axis. The long axis of bundle sheath cell is parallel to the vein axis and perpendicular to the mesophyll cell. LT = Leaf thickness; IVD = Inter-veinal distance; TML = Total mesophyll length in inter-veinal space; VW = Vein width; VH = Vein height. (B) Mesophyll and bundle sheath cell parameters, measured along X, Y, and Z. MCL = mesophyll cell length; MCH = mesophyll cell height; MCW = Mesophyll cell width; and BSCW = Bundle sheath cell width; BSCH = Bundle sheath cell height; and BSCL = Bundle sheath cell length.

The major anatomical features of a C3 rice leaf are shown in Fig 1A followed by the 3D description of the mesophyll cells and bundle sheath cells in Fig 1B. This diagram also explains the three growing axes of leaves and the cells: leaf lateral or the margin-to-margin axis (X), leaf abaxial-adaxial axis (Y), and leaf longitudinal or the proximo-distal axis (Z). The figure also shows the correct orientations used for leaf sectioning and the position of different cells inside the rice leaf.

The definitions of the anatomical traits used in the text are as follows:

***Leaf traits*:**
1 LT: Leaf thickness measured by drawing a straight line from the top of the upper epidermis to the bottom of the lower epidermis at both sides of the small veins, avoiding the uneven thickening caused by the extra stone cells and bundle sheath cells above the veins.

***Vein traits*:**
2 VD: Vein density or the number of veins in 1 mm of leaf lateral space.
3 IVD: Inter-veinal distance measured from the center of one minor vein to the center of the next minor vein.
4 TML: Total mesophyll length that fill the inter-veinal space (mesophyll cell length x mesophyll cell number in between two adjacent minor veins).
5 VW: Vein width (minor veins) or the vein diameter measured along leaf lateral (X) axis.
6 VH: Vein height (minor veins) or the vein diameter measured along the leaf ab-adaxial (Y) axis.

***Mesophyll cell traits*:**
7 MCN: Number of mesophyll cells present in between two adjacent minor veins.
8 MCL: Mesophyll cell length or the dimension of mesophyll cells measured along the X axis, as viewed in leaf transverse section.
9 MCH: Mesophyll cell height measured along the Y axis, as viewed in leaf transverse section.
10 MCW: Mesophyll cell width measured along the Z axis, as viewed in leaf longitudinal section.
11 LB_MC_: Mesophyll cell lobing, ratio of the actual periphery of a cell to the periphery of the cell circumscribed area [22] as viewed in leaf transverse section.

***Bundle sheath cell traits*:**
12 BSCN: Number of bundle sheath cells surrounding a minor vein. Extra cells out of this circle, generally known as the ‘bundle sheath cell extensions’, were not included in the scoring.
13 BSCL: Bundle sheath cell length measured along the Z axis, as viewed in leaf longitudinal section.
14 BSCH: Bundle sheath cell height measured along the Y axis, as viewed in leaf transverse section.
15 BSCW: Bundle sheath cell width measured along the X axis, as viewed in leaf transverse section.

### Statistics

One-Way Analysis of Variance (ANOVA) was carried out to test the significance of any variation in the leaf characters. Cluster analysis of species based on leaf morphology and ANOVA of different leaf anatomical traits were made using SAS version 9.1 [27] and pair-wise comparison of the mean was performed using Duncan’s Pair-wise Comparison test. Correlation values are corrected for the phylogenetically related species using phylogenetic independent contrast method [28] by using the “pic” function in “ape” package in R [29]. Phylogenetic signal in the trait values was checked by using phylosig function of phytools package [30] using K method [31] and also by checking the correlation between the phylogenetic distances between *Oryza* species to the corresponding differences in values of the traits.

### 3D modeling

Polarized leaf anatomical values were merged proportionately to construct 3D graphics that showed how the cells are actually arranged in the middle of a leaf. Mesophyll cells were drawn by adjusting the width, height, and depth of an oval shape. Similarly, bundle sheath cells were drawn by adjusting the width, height, and depth of a cylindrical shape along the X, Y and Z axes. Vein width and height were also set by adjusting the width and height of a circle. A smooth or wavy outline was selected to represent cellular lobing.

### Phylogenetic analysis

A rice phylogenetic tree was built by concatenating gene sequences of *Adh1* and *Adh*2 (S8 Table). The coding sequences (CDS) of ADH1 and ADH2 were concatenated for each rice species and used as input sequences for the phylogenetic tree reconstruction using the web tool suite “phylogeny.fr” [32]. In brief, the input sequences were first multiply aligned using the software MUSCLE [33] and then aligned positions were annotated using Gblocks software [34] with settings for a less stringent selection (allow smaller final blocks and allow less strict flanking positions). The Gblocks-annotated alignments were used for phylogeny calculation based on a maximum-likelihood method using PhyML software [35] with 100 bootstraps. The phylogenetic tree was drawn and edited using MEGA 6.06 software based on the phylogenetic tree data that resulted from PhyML.

### Ancestral state reconstruction

The ancestral state was reconstructed for 13 leaf characters using Mesquite 2.75 software [36]. For this, each of the morphological and anatomical data was grouped into three equal portions with defined minima and maxima to determine the high, low, and intermediate values for each trait. The phylogenetic tree, created using PhyML, was used as a backbone to obtain the transition parameters for ancient and recent state reconstruction using parsimony analysis [37]. Mesquite software analyzes the character state at the terminal taxa and attempts to reconstruct the ancestral state at each node and finally reconstructs the character history. This software represents graphically the history of character evolution and displays it as a phylogenetic tree.

## Results

### Leaf morphology

**Fig 2:**
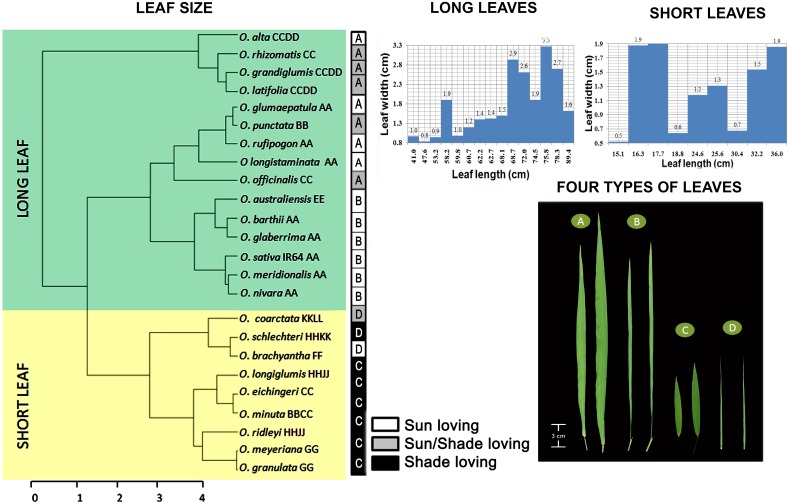
Leaf shape diversity in *Oryza*. Cluster analysis of species based on leaf blade length and leaf blade width generates two distinct groups of long and short leaved species, represented with a green background for long leaves and a yellow background for short leaves. Each species name is further accompanied with its genome type as mentioned in the text. The bar graphs show leaf blade width of long and short leaves separately to show the narrow and wide types. Representative photos of four leaf types in *Oryza* are shown as A = Long-wide (Lw), B = Long-narrow (Ln), C = Short-wide (Sw), and D = Short-narrow (Sn) leaf. Respective leaf types of *Oryza* species are mentioned in the black and white shaded boxes together with their preferred growing habitat, i.e., sunny/shaded. Notably, majority of the species in the first cluster are sun loving, whereas most of the species in the second cluster are shade loving. This suggests that sun-loving species mostly have long leaves whereas shade-loving species generally have short leaves.

Distinct differences were observed in *Oryza* leaf shapes. The leaf blade length (LL) varied approximately six-fold among the species, ranging from 15 to 89 cm. A SAS based cluster analysis, taking leaf blade length and width both together as covariance, distinctly separated the short and long leaved species in two groups (Fig 2). The range of leaf blade length of short leaves was 15.1–36.0 cm whereas, the range of the long leaves was 41.0–89.4 cm. Two graphs were plotted further to show the range of the leaf width separately for short and long leaves, taking leaf blade width as a function of the leaf blade length. This clearly showed further scope to classify the sub-groups of the main tree in narrow and wide categories. A code bar was finally added adjacent to the tree to show the specific types of leaves and also the growing habitat of each species [4], to relate the effect of growing habitat to the leaf morphological types in rice. The general appearances of these four types are shown in Fig 2 as: A for the Long-wide (Lw), B for the Long-narrow (Ln), C for the Short-wide (Sw) and D for the Short-narrow (Sn) types. The IR64 leaf (41 cm x 0.97 cm) falls under the Long-narrow category. Leaf blade length and width values of all the species are given in S1 Table.

### Leaf anatomy

**Fig 3:**
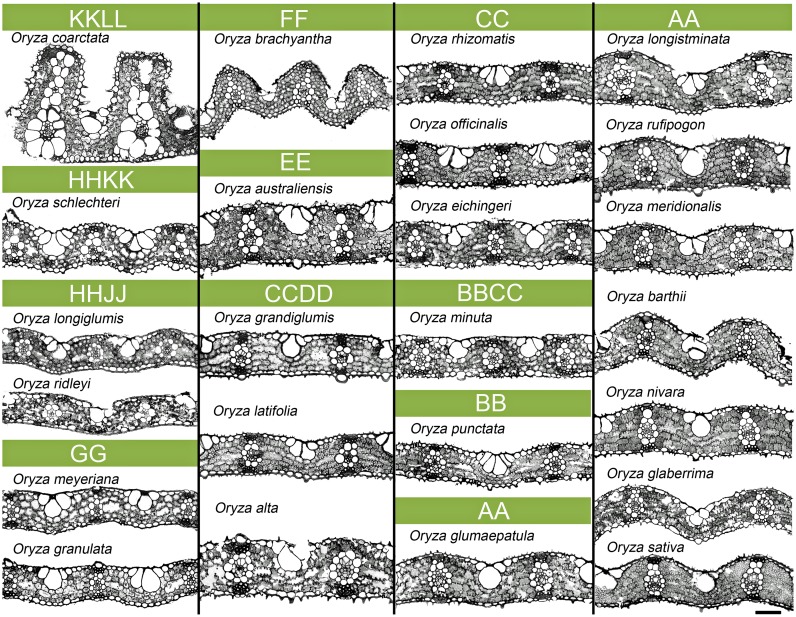
Leaf anatomical variation in *Oryza*. 2D leaf anatomical images as viewed in transverse sections. Cell types and the arrangement of cells are as described in Fig 1. Significant variation is noticed for mesophyll cell, bundle sheath cell, and vein size and shape (detailed quantification is given in S2–S5 Tables) in the *Oryza* family. *Oryza coarctata* and *O*. *australiensis* have the thickest leaves among the species. In contrast, species of the GG, HHJJ, HHKK, and BB genomes have thinner leaves. In addition, the leaves of the species of the HHKK, HHJJ, and GG genomes show closer vein spacing with relatively smaller mesophyll cells. Notably, the *O*. *coarctata* leaf possesses the widest bundle sheath cell and vertically placed additional veins unique among the rest of the *Oryza* species. Scale bar = 50 μm.

The basic anatomical features of a rice leaf, such as the position of the mesophyll cells in between the veins, bundle sheath cells surrounding the veins, the presence of stone cells above and below each vascular bundle, and the presence of bulliform cells at the upper middle portion in between two adjacent veins, all remained consistent for all the wild and cultivated species (Fig 3). An *Oryza* leaf typically contains 7–8 inter-veinal mesophyll cells and 12–13 bundle sheath cells surrounding any minor vein (general observation). Overall, the transverse sections of *Oryza* leaves appeared very diverse (Fig 3) in terms of leaf thickness, epidermal curvature, bulliform size, vein spacing, mesophyll size and number, and bundle sheath cell size and number, and varied significantly (*P*<0.001, S2–S5 Tables) among the species. Here, in particular, we have focused on quantifying the structural variation of mesophyll cell, bundle sheath cell, and vein—the three major structural elements of an *Oryza* leaf. All traits were compared to the traits of one of the most popular cultivated rice mega varieties (IR64), which belongs to *Oryza sativa* that has the most recently evolved AA genome.

### Leaf thickness

Leaf thickness (LT) varied widely from 53.7 to 375.7 μm (S2 Table) and showed 104% natural diversity compared to IR64 (LT = 74 μm), even with the exclusion of the extremely thick leaves of *O*. *coarctata*. Thick leaves were found to be supported by veins with wider diameter as in *O*. *coarctata* and *O*. *australiensis* (LT = 375.7 and 116.8 μm; VW = 36.5 and 31.9 μm; VH = 42 and 37.5 μm, respectively), whereas thin leaves were supported by narrow veins as seen in the leaves of *O*. *ridleyi*, *O*. *granulata*, and *O*. *punctata* (LT = 74.8, 61.1, 53.7 μm; VW = 22.5, 23.2, 17.1 μm; VH = 29.5, 25, 20.7 μm, respectively (S3 Table).

### Vein related traits

Generally, *Oryza sativa* has a vein density of 5 veins mm^-1^ leaf width (S3 Table). Among the wild species, VD ranged from 3.8 to 6.6 veins mm^-1^ (S3 Table). *O*. *brachyantha* (VD = 6.6 veins mm^-1^) and some other wild species such as *O*. *schlechteri*, *O*. *longiglumis*, and *O*. *ridleyi* (VD = 6.4, 6.2, 6.2 veins mm^-1^, respectively) possessed a significantly higher number of veins compared to the rest of the rice species and are referred to here as high vein density (*HVD)* species. This increase in vein number was accompanied by a subsequent reduction in inter-veinal total mesophyll length (TML) of up to 53% in *O*. *granulata* (S3 Table). In contrast, the largest TML was found in *O*. *glaberrima* (TML = 231.3 μm), which indeed had a low VD of 5.0 veins mm^-1^. The species *O*. *glumaepatula* surprisingly had thick veins (VW = 40.06, VH = 44.7 μm, S3 Table) but did not have thick leaves.

### Mesophyll cell related traits

**Fig 4:**
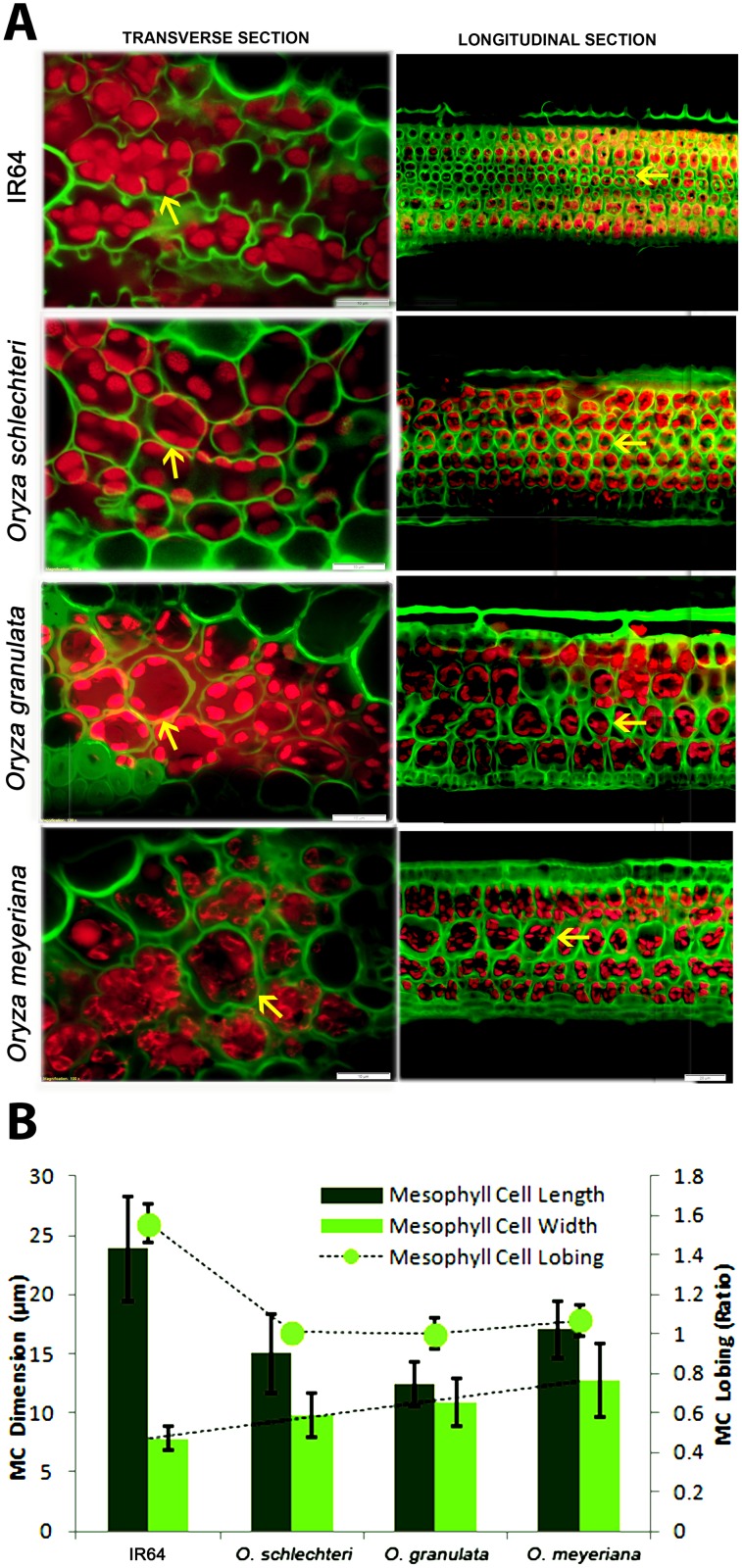
Variation in mesophyll cell size and lobing. (A) Leaf transverse sections and longitudinal section to show the mesophyll cell length, mesophyll cell lobing and mesophyll cell width in IR64 and three wild rice species: *O*. *schlechteri*, *O*. *granulata*, and *O*. *meyeriana*. The lobed/smooth line of the mesophyll cell wall (arrows) is false colored in green that is visible as a result of auto-fluorescence of the wall components. Scale bars show 10 μm distance for transverse sections and 20 μm distance for the longitudinal sections. (B) The graph shows the quantitative values (average ±SD) of MCL, MCW, and LB_MC_ (secondary axis). MCL = mesophyll cell length, MCW = mesophyll cell width, LB_MC_ = mesophyll cell lobing.

Mesophyll cells are the major chloroplast containing green cells inside a rice leaf and are the main sites of photosynthesis. All of the mesophyll cell structural traits varied significantly (*P*<0.001, S4 Table) among the species. We observed two distinct types of mesophyll cell in rice: termed here as Type-A that is without cell wall lobing and Type-B that is with cell wall lobing. The first type comprises *O*. *schlechteri*, *O*. *longiglumis*, *O*. *ridleyi*, *O*. *meyeriana*, and *O*. *granulate*, which had no or very sparsely lobed walls (LB_MC_ = 1–1.1, significantly different from other species, *P*<0.001) with generally smaller cells (except *O*. *ridleyi*). The second type has a profusely lobed cell wall (a maximum of LB_MC_ = 1.7 as in *O*. *meridionalis*) and generally the cells appeared larger in transverse section. Notably, three ‘Type-A’ species (*O*. *schlechteri*, *O*. *meyeriana* and *O*. *granulata*) possessed wider mesophyll cells (MCW = 9.7, 12.7, and 10.9 μm, respectively, Fig 4A and 4B) in longitudinal axis. In terms of the number, none of the wild species had more mesophyll cells in between two veins than in IR64; although two species (*O*. *alta* and *O*. *glaberrima*) had laterally larger mesophyll cells (MCL = 36.9 and 32.4 μm respectively). The minimum number of mesophyll cells (MCN = 4 to 5) was found in *O*. *coarctata*, *O*. *grandiglumis*, and *O*. *minuta*.

### Bundle sheath cell related traits

The minor veins in the IR64 leaf are typically surrounded by 12–13 bundle sheath cells (S5 Table). An inverse trend was observed between the number and size of the bundle sheath cells. Larger bundle sheath cells were found to be fewer in number, surrounding a vein (S5 Table). *Oryza coarctata* was found to have a strikingly wide bundle sheath cell (BSCW = 45 μm and BSCH = 29 μm) uniquely different from any other *Oryza* species.

### Anatomical variation is independent of morphological types

**Fig 5:**
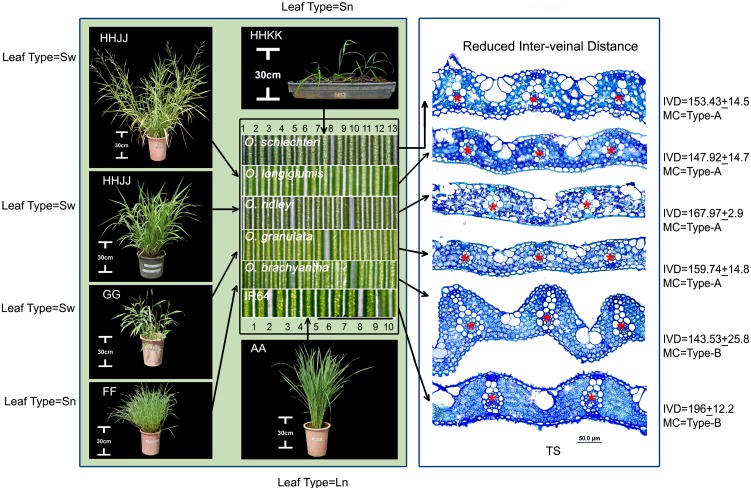
High vein frequency is conserved in closely related wild rice but not dependent on the leaf morphological types. Leaf surface images at the middle of the figure, show increased vein density (white parallel bands) in morphologically diverse leaves (see plant images) of closely related species of HHKK, HHJJ, GG, and FF genomes. Numbers, at the top and below these leaf surface images represent the vein number at 2mm space in case of *O*. *schlechteri* (one of the high vein density species) and *O*. *sativa* IR64. Scale bar under the leaf surface image = 1 mm. Positions of the veins are marked by red stars (*) in the leaf transverse section (TS) at the right, confirmed that the increased vein frequencies are due to reduced inter-veinal distance (IVD). Leaf types (Sw/Sn), mesophyll (MC) types (A/B), and inter-veinal distance (IVD) are indicated. Sw = Short-wide leaves, Sn = Short-narrow leaves.

**Fig 6:**
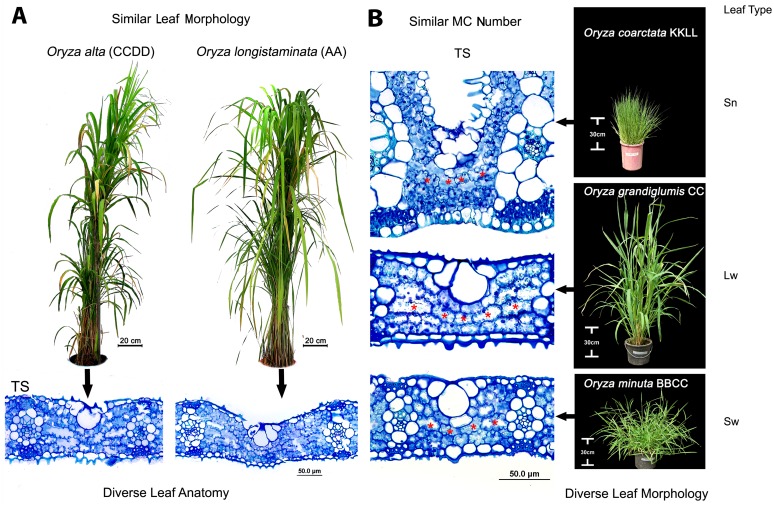
Similar leaf morphology or anatomy appears in diverse rice genomes. (A) *Oryza alta* (CCDD) and *O*. *longistaminata* (AA) show similar Long-wide leaf morphologies but quite different in their leaf anatomies. (B) Similar mesophyll cell numbers (4–5, marked by the red stars in leaf transverse sections) appear in *O*. *coarctata*, *O*. *grandiglumis*, and *O*. *minuta* (KKLL, CC, and BBCC genome respectively) in spite of their markedly different leaf types. Lw = Long-wide, Sn = Short-narrow, Sw = Short-wide.

We found that the total number of veins (counted from leaf margin to margin in the widest part in the middle of a leaf) is strongly (r = 0.9, *P*<0.005) related to leaf width (S1 Fig). The relation is strong even after the phylogenetic correction (r = 0.84, *P*<0.005, S9 Table for phylogenetic signal). But surprisingly, an increased number of veins in a unit leaf length (VD) were found not to relate to any one morphological type. For example, the high vein density (*HVD*) character was found in wide leaves of *O*. *longiglumis*, *O*. *ridleyi*, *O*. *meyeriana*, and *O*. *granulata* (LL = 16 to 32 cm x LW = 1.5 to 1.9 cm) as well as in narrow leaves of *O*. *schlechteri* and *O*. *brachyantha* (LL = 30.4 cm x LW = 0.7 cm, and LL = 18.8 cm x LW = 0.7 cm, respectively, Fig 5). The two narrow-leaved *HVD* species further differed in their mesophyll characteristics (Type-A in *O*. *schlechteri* and Type-B in *O*. *brachyantha*). Therefore, in terms of mesophyll cell structure, *O*. *schlechteri* is more similar to the broad-leaved *HVD* species, whereas *O*. *brachyantha* is more similar to the rest of the *Oryza* species carrying Type-B MC, irrespective of having narrow or wide leaf morphologies. Similarly, the long wide leaves of *O*. *alta*, *O*. *grandiglumis*, *O*. *longistaminata O*. *glumaepatula*, and *O*. *rufipogon* is diverse in several anatomical aspects (Fig 3 for 2D anatomies, examples of *O*. *alta* and *O*. *rufipogon* are shown in Fig 6A). Following this trend, fewer mesophyll cells (4–5 in number) in between minor veins in *O*. *coarctata*, *O*. *grandiglumis*, and *O*. *minuta* are independent of their very different leaf shapes (Fig 6B).

### Construction of 3-dimensional leaf anatomical model

**Fig 7:**
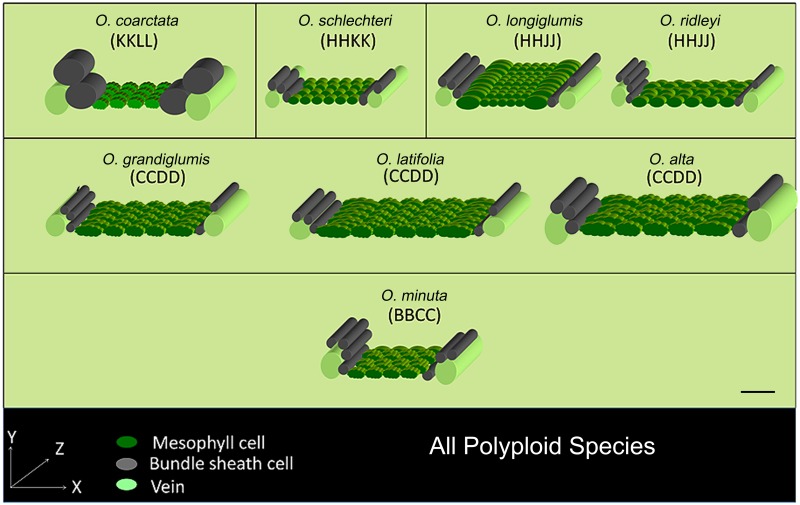
3-Dimensional anatomical models of plolyploid *Oryza* rice leaves. The 3D models were constructed by combining the measurements taken separately along the three growth axes (X, Y, and Z) for all polyploid *Oryza* species. Note that, when considering the perpendicular spatial positioning of the mesophyll cell and bundle sheath cell to each other; X, Y, and Z represent length, height, and width of mesophyll cell, and the width, height, and length of bundle sheath cell respectively. Mesophyll cells are colored in green to represent the main photosynthetic tissue, veins are colored in light green, and bundle sheath cells are colored gray. Wavy surfaces are applied to the mesophyll cell boundaries with a degree of lobing value more than 1.1 (Type-B mesophyll cell). Type-A mesophyll cell is shown with smooth wall structure as in *O*. *schlechteri*, *O*. *longiglumis*, *O*. *ridleyi*. Genome types are as described in the text. A calibration scale of 50 μm is provided.

**Fig 8:**
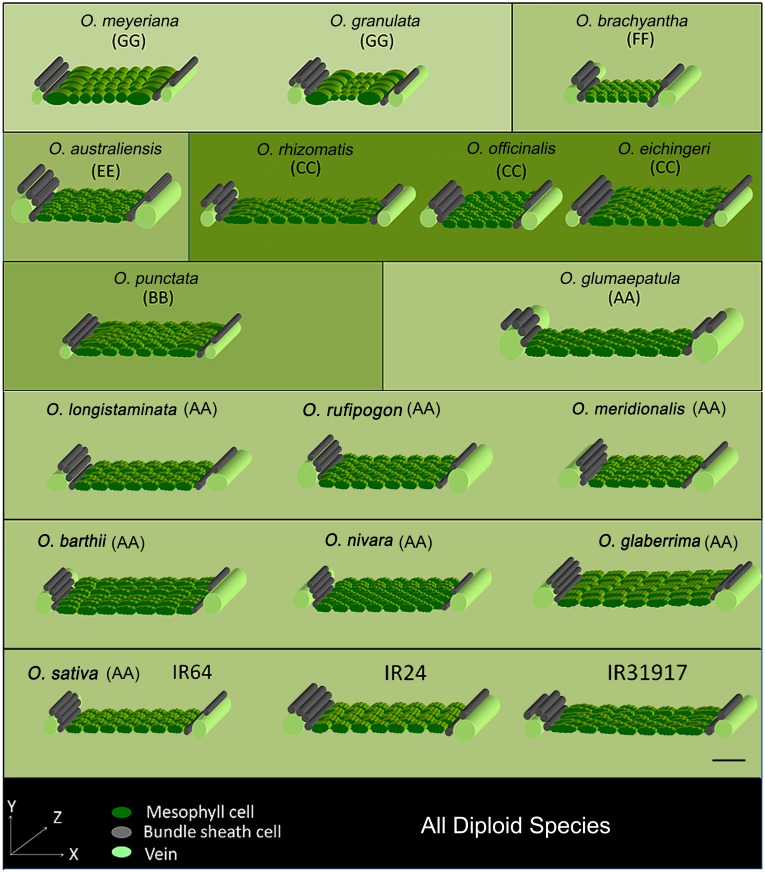
3-Dimensional anatomical models of diploid *Oryza* rice leaves. 3D models describe the variation of rice leaf cellular structure of all diploid *Oryza* species; especially describing the characters of mesophyll cells, bundle sheath cells and veins. The construction of the 3D models is as described as in Fig 7. Type-A mesophyll cell is shown with smooth wall structure as in *O*. *meyeriana* and *O*. *granulata*. Three *Oryza sativa* cultivars IR64, IR24 and IR31917 are shown to provide a broad idea about the leaf anatomy of present day cultivated rice species. Genome types are as described in the text. A calibration scale of 50 μm is provided.

3-Dimensional (3D) leaf anatomy models represent the actual volume and orientation of the mesophyll and bundle sheath cells in rice leaves. These models were constructed by combining all the cellular dimensions measured along the lateral and longitudinal directions, and then serially orienting the appropriate number of cells (Figs 7 and 8). We noticed that the long axis of the bundle sheath cell is actually oriented perpendicular to the long axis of the mesophyll cell. Hence, although the length of the mesophyll cell can be viewed in transverse section, the length of the bundle sheath cell can be viewed only in leaf longitudinal and paradermal section. Due to the perpendicular placement of mesophyll cells and bundle sheath cells to each other; the lateral, vertical, and longitudinal axes of the leaf actually describe the length, height, and width of the mesophyll cell but, oppositely, the width, height, and length of the bundle sheath cell. For a better illustration, a wavy border was applied to the mesophyll cell where a lobing value was greater than 1.1 (significantly different from the rest of the species, *P*<0.005, S4 Table). Veins were also drawn as cylinders at both ends of the 3D models. Along with IR64, two other popular *O*. *sativa* cultivars (IR24 and IR31917) were also modeled as checks (values are shown in S6 Table). These models provide an overall comparative view of the leaf cellular arrangement in rice and its wild relatives.

### Assessment of ancestral leaf characters

**Fig 9:**
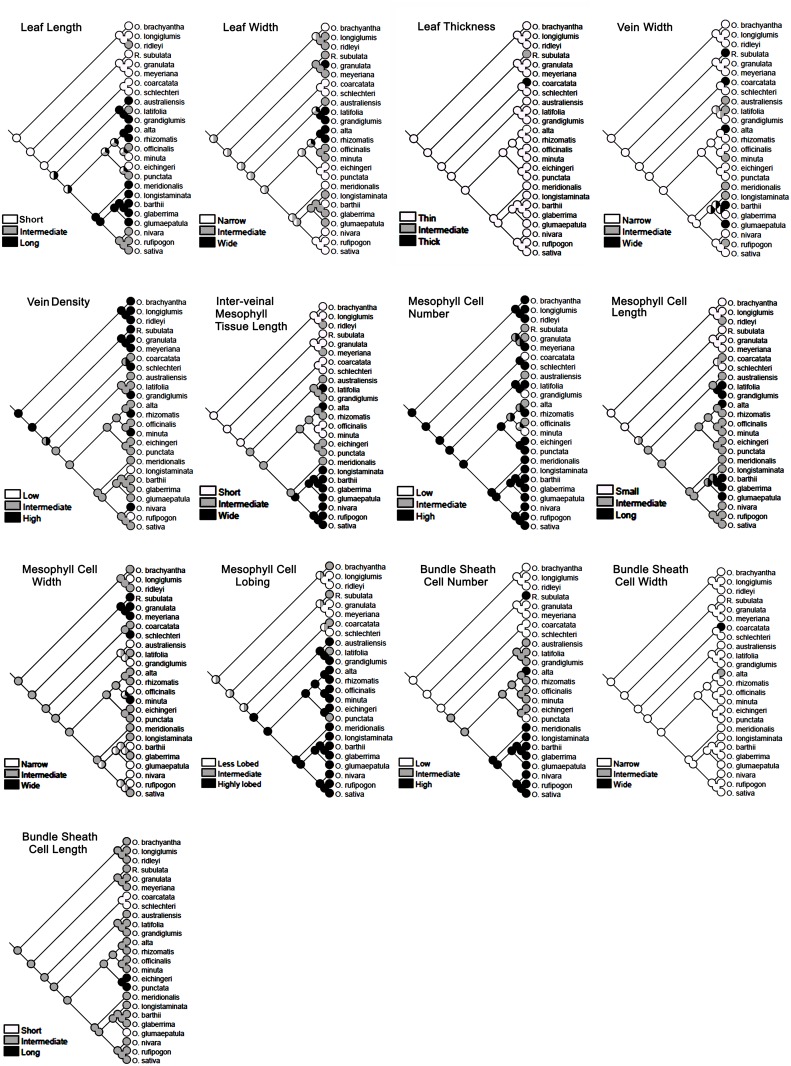
Ancestral state reconstruction of leaf morphology and anatomy traits. Historical analysis of a total of 13 leaf traits, taking all the *Oryza* species, confirm that small leaf, high vein density, shorter inter-veinal mesophyll area, smaller-sized mesophyll cells, and fewer number of bundle sheath cells surrounding a vein, are the primitive leaf characters in rice. Likewise, a wider inter-veinal mesophyll area, highly-lobed mesophyll cells, and increased bundle sheath cell numbers are advanced characters in cultivated rice leaves. *Rhynchoryza subulata* was used as an out-group.

**Fig 10:**
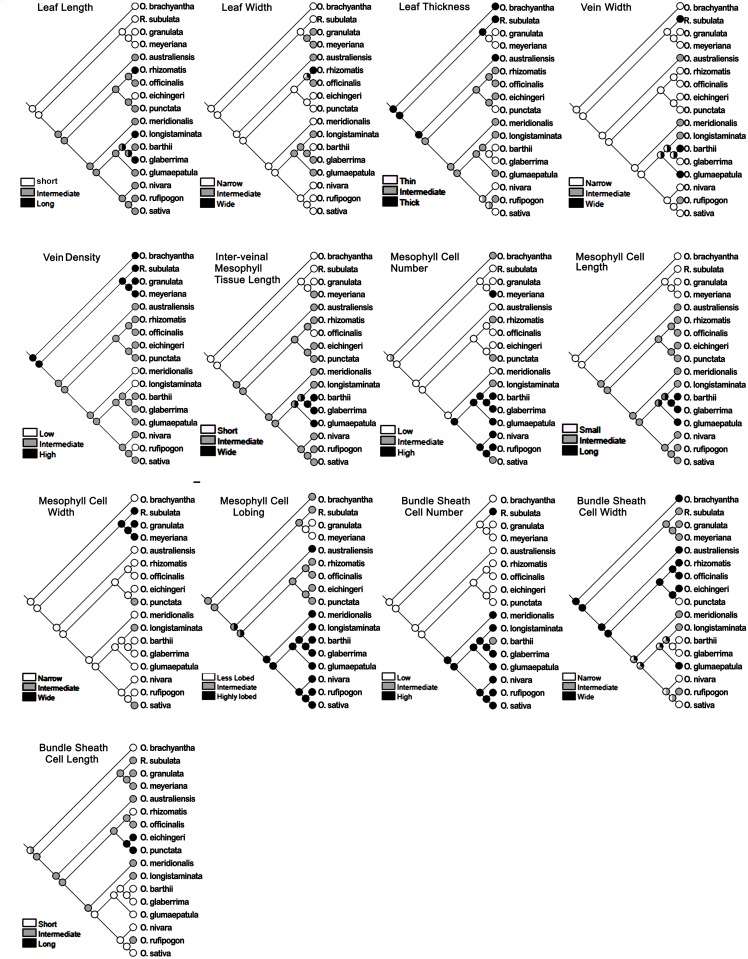
Ancestral state reconstruction of leaf morphology and anatomical traits in diploid *Oryza* species. The history of the evolution of leaf traits in diploid species confirms the increase in the inter-veinal mesophyll area, mesophyll number, mesophyll length and bundle sheath cell number over time in rice. Leaf thickness and bundle sheath cell width also appear to be reduced in the recently evolved rice species.

The ancestral state was reconstructed for leaf blade length, leaf blade width, leaf thickness, three vein characters, four mesophyll characters, and three bundle sheath cell characters for all the *Oryza* species (Fig 9), and also separately for all the diploid species (Fig 10). The two nuclear genes *Adh1* and *Adh2* were used here to construct the phylogenetic tree of *Oryza* family that has been used further as a backbone for the leaf ancestral character tracing. These two genes are widely used in grass phylogenetic studies [7, 38, 39]. *Rhynchoryza subulata* was used as an out-group. The phylogenetic tree topology (S2 Fig) showed monophyly of the species with nested small groups formed by closely-related genomes. After comparing the distribution of character states in the terminal taxa on that rice phylogenetic tree, the evolutionary history of each character was traced using character ancestral state analysis using parsimony. The ancestral state reconstruction clearly showed that small leaves, more vein number, reduced inter-veinal length, less mesophyll cell length, and fewer bundle sheath cells were the primitive characters in rice (Fig 9). Characters like leaf width, and mesophyll cell lobing might have evolved from an ancestor having an intermediate value for these traits (Fig 9), though, a much more clear trend of evolving highly lobed mesophyll cells from the less lobed mesophyll cell has been observed in case of the diploid species (Fig 10). Similarly, the trend of evolving thinner leaves from thicker leaves, and smaller bundle sheath cells from larger bundle sheath cells has been observed in case of the diploid species (Fig 10). The history of some of the traits such as leaf thickness, vein width, bundle sheath cell width, and bundle sheath cell length could not be resolved due to the general homogeneity of these traits in *Oryza*.

## Discussion

Leaf is a multicellular determinate organ and is controlled by regulation of complex genetic networks, interaction between cell cycle and cell enlargement during its developmental stages, according to neo cell theory [40, 41]. Generally, the leaf meristem develops from the flanking region of the shoot apical meristem (SAM). Recruitment of founder cells is cued by down-regulation of *KNOTTED-1 like homeobox* (*KNOX*) *genes*. This is followed by determination of the leaf polarity; where class III homeodomain leucine zipper protein (*HD-ZIP III*), determines the adaxial leaf surface and *KANADI* (*KAN)* and the *YABBY* gene family together with the *AUXIN RESPONSE FACTORS* (*ARF3* and *ARF4*) determine the leaf abaxial-polarity [42]. To date, studies on leaf anatomy have been largely focused on the eudicot leaf especially in Arabidopsis [43–45], specifying cell polarity [46, 47], cell patterning [48, 49] and cell shape development [50–52]. Transcriptome sequencing has been recently applied in maize to gain an in-depth knowledge of the regulation of gene expression during leaf development [53–55]. Recently, a *WUSCHEL-related homeobox* gene has been reported to work antagonistically with *YABBY* to control leaf width in rice [56]. A number of anatomical factors like mesophyll cell structure, mesophyll cell conductance and chloroplast area in mesophyll exposed to the CO_2_ in intercellular space control the functional variation of leaves [57], but a clear knowledge of factors that regulate rice leaf anatomy is still obscure and limited by the identification of structurally-diverse rice species.

### Leaf anatomical diversity in rice is under genetic control

Distinct variation in the leaf cellular structures of species of different rice genomes, that contain different genetic blueprint, suggests genetic control over leaf anatomical modules in rice. At the same time, a number of leaf anatomical traits are found to be quite conserved within and across the genomes (S2 Fig). For example, the close vein spacing of Type-A mesophyll cell, in all the known existing species of closely-related GG, HHJJ, and HHKK genomes, provides clear evidence of the genetic conservation of the trait or phylogenetic inertia (Fig 5). Similarly, the presence of Type-B mesophyll cell in the rest of the species also suggests a strong genetic control over this trait that is possibly also phylogenetically conserved. The uniformity of leaf anatomy in the biggest family AA is quite expected as its members are genetically less diverse [58–60] and, therefore, possibly contain similar anatomy-controlling gene pools across the species. However, the cellular structure of leaves of *O*. *punctata* (BB genome) looks quite similar with that of the species in the AA genome (Figs 2 and 8). This indicates the conservation of shared anatomical genes in these two phylogenetically close genome types. Interestingly, most of the anatomical traits (except the Type-B mesophyll cell) are not conserved starting from the EE genome to the BBCC genome (S2 Fig), which probably indicates a buffer period in rice history, where preferable genetic recombination for leaf characters had been tried to obtain final optimum leaf structure in cultivated rice.

It has been reported earlier that an increase in vein number per unit area in rice leaves was due to a change in vein diameter or due to the reduced size of other cells as an immediate compensatory mechanism [20, 61]. Here we demonstrate that the increase in vein number in *Oryza* can also be due to an actual change in the number of mesophyll cells rather than just having a change in cell size (S4 Table). This confirms independent regulatory mechanisms for cell division and cell expansion exist in the *Oryza* genus.

Interestingly, a number of species showed very thick veins compared to IR64 (*O*. *coarctata*, *O*. *brachyantha*, *O*. *australiensis*, and *O*. *glumaepatula*) though their functionality still needs to be resolved. Larger mesophyll cells in the wild species *O*. *glaberrima* and *O*. *alta* without a compensatory change in the number of mesophyll cells could be a useful trait to engineer into cultivated rice as a means to enhance photosynthesis and yield. Larger mesophyll cells might provide increased mesophyll cell surface area to access more intercellular CO_2_ and also can have more chloroplasts inside the cell, which is quite beneficial for increasing photosynthesis [22].

### 3D model of key anatomical features of rice

Conventional microscopic studies generally describe leaf structure in two dimensions only. However, the exact nature of a cell’s structure is best achieved using 3-dimensional analysis. 3D leaf imaging through magnetic resonance imaging [62] and X-ray tomography [63] as well as 3D graphics using multiphoton laser scanning microscopy and X-ray computed laminography have been used in Arabidopsis [64] and tomato leaves [65] but applying these techniques on rice still remains challenging. The 3D leaf models are constructed to facilitate the analysis of cell number, type, volume, and orientation (Figs 7 and 8) of the major cells inside a rice leaf. These models help us to gain new insights into leaf structural variation that possibly accounts for differences in their physiology too. These 3D models together with additional 2D features and leaf morphologies give a clearer idea of the key leaf structural features for each rice genome and are also used to identify novel evolutionary trends.

Generally, the species of the AA and BB genomes characteristically retain wide vein spacing, linked by 7–8 elliptical, laterally-flattened, and profusely-lobed Type-B mesophyll cells, arranged at right angles to the vein in both long-narrow and long-wide leaf types. A hollow tube was formed by the stacking of cylindrical bundle sheath cells, joined end to end, to wrap the vein throughout the leaf length.

The curvy adaxial surface of the hard, needle-shaped, leaves of *O*. *coarctata* (KKLL) is patterned with dorsal alternate ridges, which are supported by extra veins, bundle sheath cells, and mesophyll cells (Fig 3). This makes the leaves of *O*. *coarctata* approximately three-fold thicker than the leaves of *O*. *sativa*. This species possesses relatively small Type-B mesophyll cells but has the shortest but widest bundle sheath cell.

The species of the HHKK, HHJJ, and GG genomes together contained both short-narrow and short-wide leaves. These species characteristically possess relatively smooth walled (lobeless), small globular Type-A mesophyll cells and an increased vein number supported with reduced inter-veinal mesophyll length (TML).

The increased vein density with reduced TML was maintained in the leaves of *O*. *brachyantha* in the FF genome. Mesophyll cells were still smaller, but considerably lobed (LB_MC_ = 1.31, S4 Table). The lobing was either maintained or increased in the succeeding genomes. Leaves of *O*. *australiensis* of the EE genome characteristically retained markedly thicker leaves (LT = 116 μm) with reduced inter-venial distance and TML, and also showed a reduction in mesophyll cell number (MCN). The leaves of the species of the CCDD genome were easily identifiable by their very long and wide leaf shape (S1 Table). In terms of leaf anatomy, this genome possesses larger mesophyll cells (MCL = 27.4–36.9 μm, S4 Table).

A wide variation in the leaf morphology of the CC genome makes it difficult to describe a signature leaf trait for this genome. Leaves were of two different types: long-wide in *O*. *rhizomatis*, *O*. *officinalis*, and short-wide in *O*. *eichingeri* (S1 Table). The vein density varied from 4.9 to 5.6 veins mm^-1^ and IVD ranged from 181.7 to 218.6 μm, with appropriate adjustment in mesophyll cell size and vein diameter (S3 and S4 Tables). A significant reduction in mesophyll cell number (MCN = 4) was the main anatomical characteristic of the allo-tetraploid species *O*. *minuta* of the BBCC genome. *Oryza punctata* of the BB genome showed markedly thinner leaves (LT = 53.76 μm, S2 Table).

### Ancestral characters

After comparing all the 3D structures (Figs 7 and 8), we can clearly see that the first marked change in leaf anatomy during rice evolution (following a retrogressive path) was in *O*. *minuta* (BBCC) that occurred approximately before 0.4 million years [6]. This allo-tetraploid species showed relatively thicker leaves with more veins per mm of leaf width, and had 4–5 mesophyll cells in the inter-veinal region, which is significantly less than that of the leaves of rice cultivated today. These traits were more similar to that of the species of the CC and CCDD genomes, and thus retained their ancestral inheritance.

Similar leaf anatomical features, observed in related species (S2 Fig), trigger the question about the history of these characters during rice evolution. Tracing back the ancestral characters (Fig 9) clearly shows that a short leaf was a primitive character in rice. Long leaves are derived from the shorter leaves with repetitive occurrences of the short type in the recent BBCC and CC genomes as an acquired characteristic. It is known that polyploidy can affect organ size [66]. The giant long leaves observed in the allo-tetrapolid CCDD genome might be a similar polyploid effect, which was also observed in three diploid species of the AA genome (*O*. *logistaminata*, *O*. *rufipogon* and *O*. *glumaepatula*), which might be due to conservation and dominancy of similar genes. It is also possible that these giant leaves were initially evolved as a requirement for their sunny habitat, but had finally been optimized to a moderate leaf size in recently evolved rice species to optimize the stoichiometry of leaf structure and function. Our historical analysis proposes an intermediate mesophyll cell lobing type as being ancestral (Figs 9 and 10). To further confirm if the mesophyll cell lobing had really evolved from the non-lobed character, we studied the leaf anatomy of a few more *Oryza* wild relatives (called distant wild rice species because of their morphological similarity to the wild rice plant). These species possessed either of that two mesophyll cell types (S3 Fig). In *Hygroryza aristata* and *Chikusichloa aquatica*, a complete reduction of lobing was observed, whereas the mesophyll cell wall of *Luziola leiocarpa*, *Rhynchoryza subulata*, *Leersia tisseranti*, and *Zizaniopsis villaniopsis* showed varying degrees of lobing from 1.00–1.38 (S7 Table). All these species showed a reduction in either mesophyll cell number or length or both, confirming again that these characters were really primitive in the evolution of the *Oryza* leaf.

### Leaf evolution in rice

**Fig 11:**
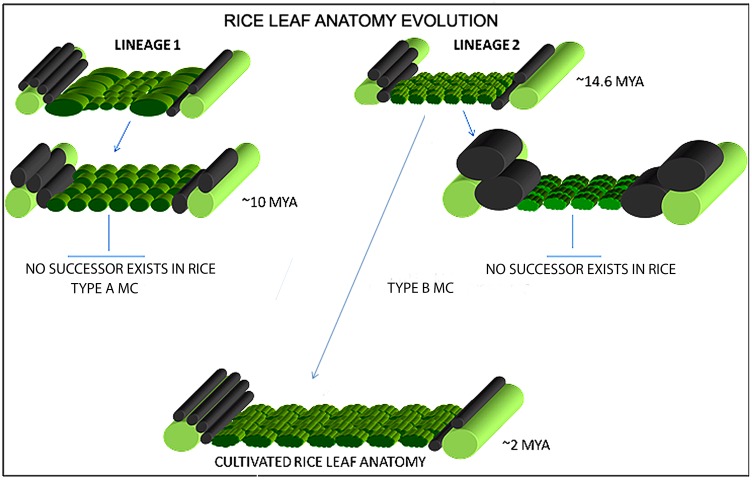
Rice leaf evolution. A two-pronged leaf evolutionary hypothesis in rice suggests that the leaf structure has possibly evolved into *Oryza sativa* leaf type by the favorable selection of one of the two possible evolutionary lines (Lineage 1 and 2). The first lineage explains the presence of Type-A mesophyll cells, which existed ~10 million years ago and has remained unchanged since the evolution of *O*. *schlechteri*. The second lineage explains a gradual modification of the ancestral Type-B mesophyll cells that lead to an overall increase in the mesophyll area between the veins and gradually evolved into the cultivated rice leaf that we see today. The probable time of evolution of a particular leaf type is shown as Million Years Ago (MYA).

The changes in leaf characters during rice evolution (S2 Fig) indicate the gradual dominance of certain anatomical characters during speciation. Our analysis suggests that Type-A and Type-B mesophyll cell structures have possibly evolved from a common ancestor even before the foundation of the *Oryza* species i.e., at least 15 million years ago which is the reported origin time of rice [6, 67]. Therefore, according to the constructed rice phylogeny (S2 Fig) and to available chronological data [6], *O*. *granulata* and *O*. *brachyantha* were the first known diploid species to have the Type-A and Type-B mesophyll cells respectively. These two contrasting mesophyll cell types were present in a number of rice species until 11 million years ago, the possible origin time of the allo-tetraploid KKLL and HHKK genomes [6] and after that the Type-A mesophyll cell has remained unchanged. A unique increase in the bundle sheath cell lateral diameter or width (BSCW), seen in *O*. *coarctata*, was also not found in any other rice species that evolved after *O*. *coarctata*. Therefore, the evolution of large bundle sheath cells can be considered as an evolutionary dead end that terminated with *O*. *coarctata*. These observations led to the hypothesis of parallel leaf evolutionary lineages in rice (Fig 11), taking *O*. *granulata* and *O*. *brachyantha* as the existing founder species of each lineage. Both lineages, with the basic difference of mesophyll cell lobing, started with a common feature of reduced total mesophyll length in between the veins; and gradually modified this to achieve increased mesophyll area by increasing mesophyll cell number and length over evolutionary time. We conclude that the first lineage had lasted for a short time span of approximately 4 million years and was lost with *O*. *schlechteri*. In contrast, gradual increases in size, lobing, and number of mesophyll cells in the second lineage ultimately led to the formation of the present-day leaf anatomy of rice. An increase in mesophyll area possibly resulted increase in photosynthetic tissue within the leaf, which eventually lead towards an improved photosynthetic efficiency in cultivated rice.

## Conclusion

This study provides new insights into rice leaf diversity, described in an evolutionary context. We have dissected rice leaf morphological and anatomical traits and reported significant leaf morpho-anatomical diversity for these traits in wild rice, particularly for the veins, mesophyll cells, and bundle sheath cells. We have concluded that leaf morphology and anatomy are not always linked and thus recombining those traits can open new horizons to engineer a new leaf types or even a C4-like rice leaf structure which requires very narrow vein spacing. Increasing rice vein density, mesophyll size, and bundle sheath cell size is also possible using the trait from appropriate wild species. Likewise, some of the *Oryza* species identified with thick leaves could be of direct interest for rice breeding. This paper presents a thorough genome-wide study and helps to identify the ancient and recently-evolved leaf characters. Finally the analysis of 3D leaf anatomical models within an evolutionary context clearly shows that the increment in the mesophyll area is the key reason behind rice leaf succession towards the present-day *O*. *sativa* leaf type. Further studies on the functional significance and genetic regulation of these anatomical traits will be an exciting avenue for future research.

## Supporting Information

S1 Fig Relation between leaf width and the total number of veins (VD x LW) in leaves of *Oryza*. (TIF)

S2 Fig Phylogenetically arranged leaf trait variation in *Oryza* species. The phylogenetic tree of *Oryza*, created using conserved nuclear sequences of *Adh*1 and *Adh*2. Aligning leaf data matrix with the tree suggests the successive changes that might have occurred in various leaf traits during *Oryza* speciation. Heat map of the leaf traits shows certain colored patches (black for low values and green for high values) suggesting cross-species conservation of similar leaf traits. For example, small leaves (1), reduced inter-veinal mesophyll cell length (2), small (3) and reduced lobed mesophyll cell (4), reduced bundle sheath cell diameter (5 and 6), more veins (1a), wider total mesophyll area (2a), mesophyll cell number (3a), increased mesophyll cell lobing (4a), and increased bundle sheath cell number (5a) show conserved characters in closely related wild/cultivated species. 3D anatomy models at the right are shown to compare the overall changes in anatomy that happened during evolution. (*Rhynchoryza subulata* was used as out group).
(TIF)

S3 Fig Leaf anatomy (2D) of distant wild rice relatives. Types and arrangement of cells as describe in Fig 1.
(TIF)

S1 Table Leaf length and leaf width of *Oryza* species. (PDF)

S2 Table Leaf thickness of *Oryza* species. (PDF)

S3 Table Vein characters of *Oryza* species. (PDF)

S4 Table Mesophyll cell characters of *Oryza* species. (PDF)

S5 Table Bundle sheath cell characters of *Oryza* species. (PDF)

S6 Table Detailed anatomical characters of three high yielding rice cultivars IR64, IR24 and IR31917. (PDF)

S7 Table Detailed anatomical characters of distant wild rice species. (PDF)

S8 Table *NCBI* accessions of the genes used in constructing the rice phylogenetic tree. (PDF)

S9 Table Phylogenetic signal in the leaf traits. (PDF)
