# Supplementary figures and images for: The Evolutionary Basis of Naturally Diverse Rice Leaves Anatomy
Source: PLoS One. 2016 Oct 28;11(10):e0164532. doi: 10.1371/journal.pone.0164532 (PMC5085062; doi:10.1371/journal.pone.0164532)

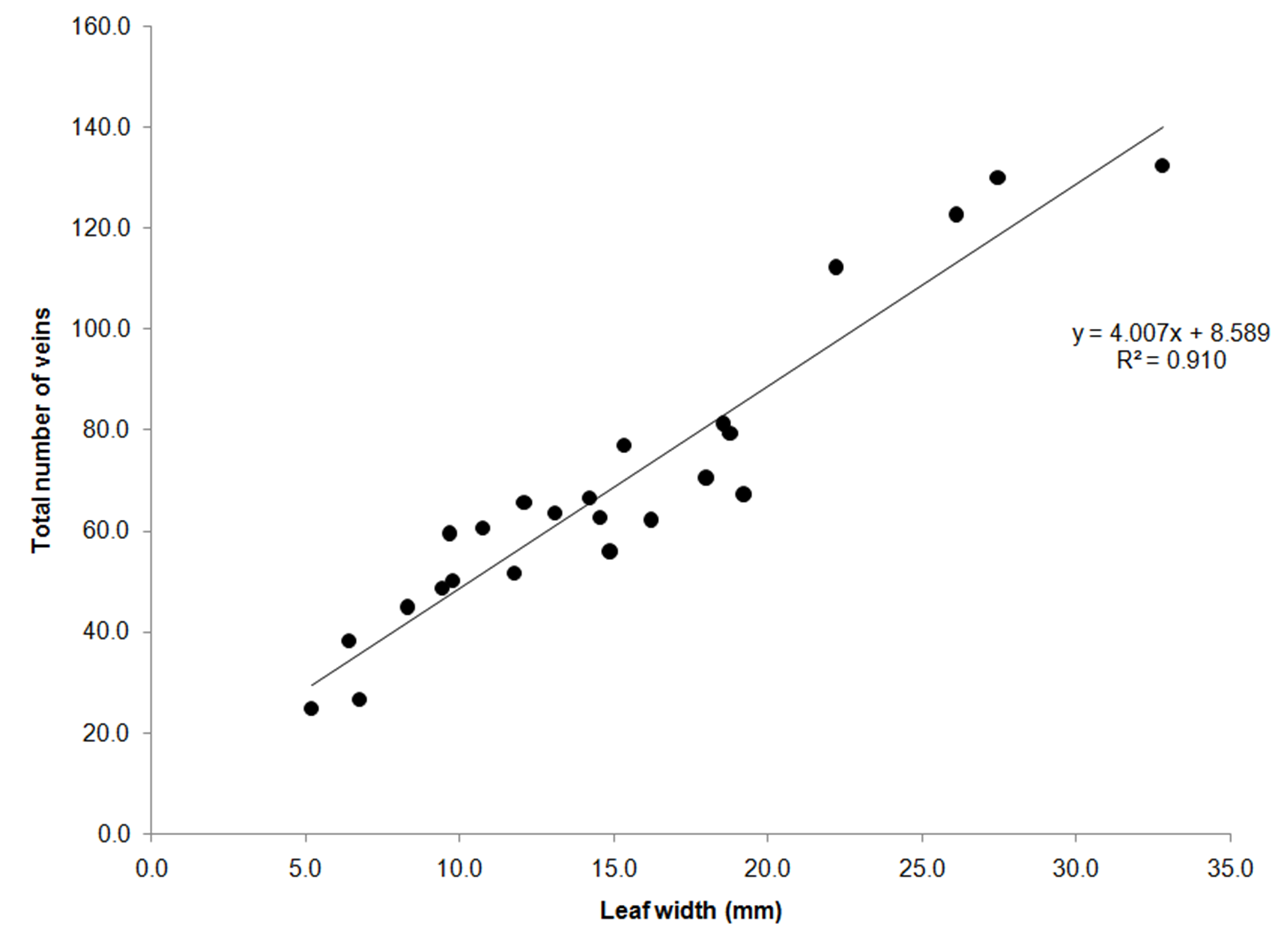

Supplement: S1 Fig — (TIF) [file pone.0164532.s001.tif]

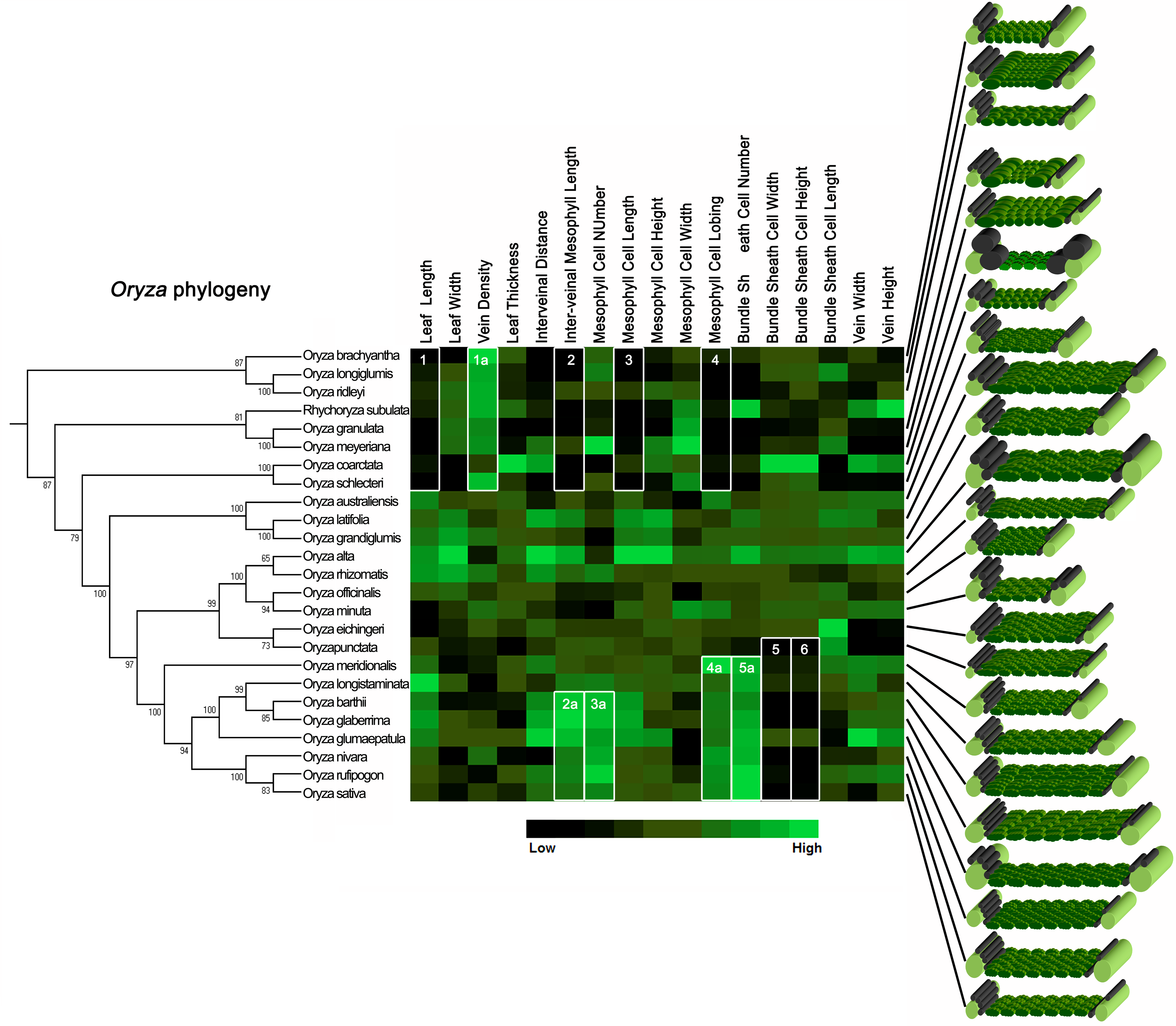

Supplement: S2 Fig — The phylogenetic tree of Oryza, created using conserved nuclear sequences of Adh1 and Adh2. Aligning leaf data matrix with the tree suggests the successive changes that might have occurred in various leaf traits during Oryza speciation. Heat map of the leaf traits shows certain colored patches (black for low values and green for high values) suggesting cross-species conservation of similar leaf traits. For example, small leaves (1), reduced inter-veinal mesophyll cell length (2), small (3) and reduced lobed mesophyll cell (4), reduced bundle sheath cell diameter (5 and 6), more veins (1a), wider total mesophyll area (2a), mesophyll cell number (3a), increased mesophyll cell lobing (4a), and increased bundle sheath cell number (5a) show conserved characters in closely related wild/cultivated species. 3D anatomy models at the right are shown to compare the overall changes in anatomy that happened during evolution. (Rhynchoryza subulata was used as out group). (TIF) [file pone.0164532.s002.tif]

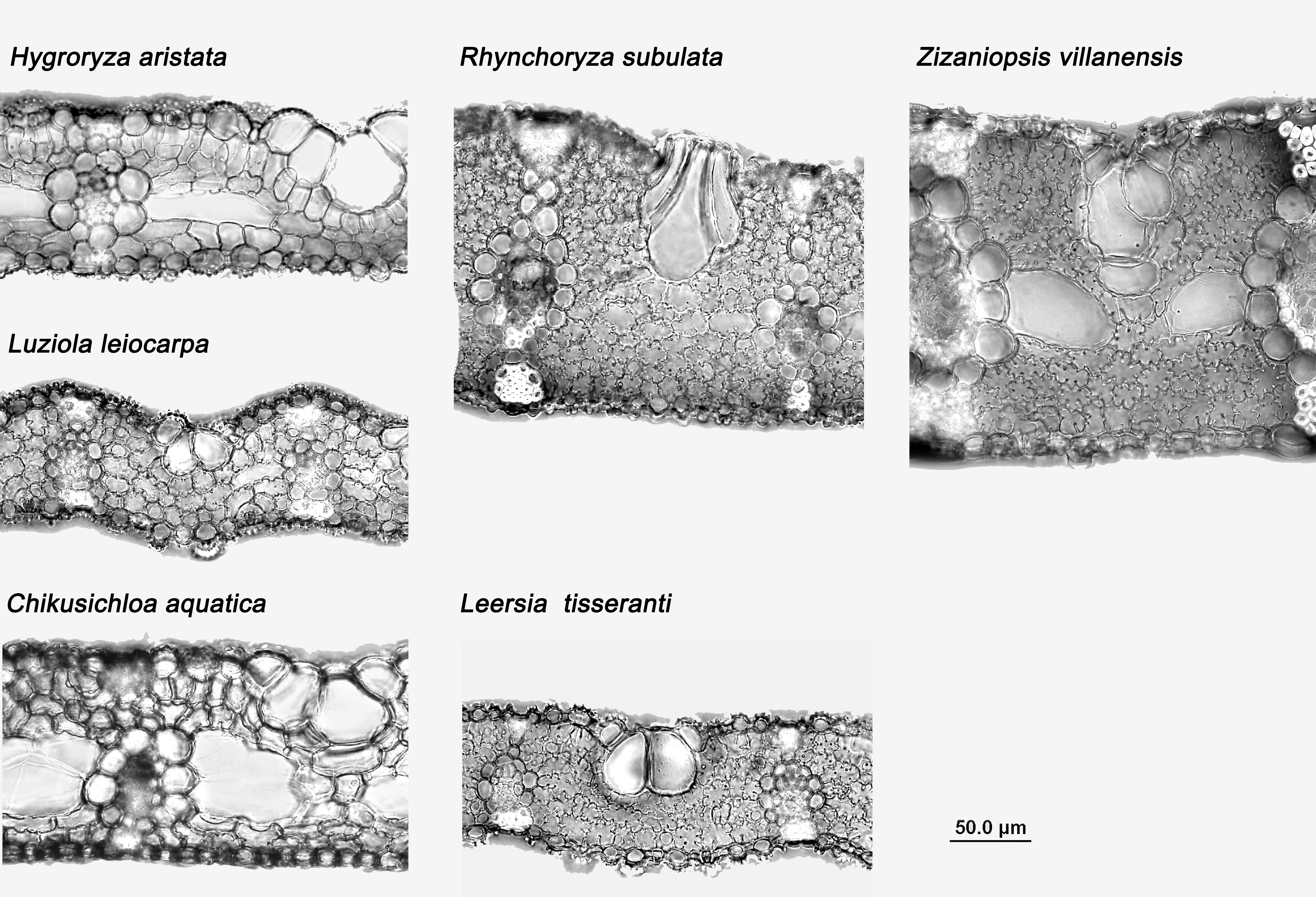

Supplement: S3 Fig — Types and arrangement of cells as describe in Fig 1. (TIF) [file pone.0164532.s003.tif]
