# Supplementary material for: The Evolutionary Basis of Naturally Diverse Rice Leaves Anatomy
Source: PLoS One. 2016 Oct 28;11(10):e0164532. doi: 10.1371/journal.pone.0164532 (PMC5085062; doi:10.1371/journal.pone.0164532)
Supplement: S1 Table — (PDF) [file pone.0164532.s004.pdf]

S1 Table. Leaf length and leaf width of *Oryza* species.

The average leaf blade length (LL) and leaf width (LW) are computed taking nine leaves per species (N = 9) from three different plants. The length is measured from base to tip and the width is measured at the middle of the leaf. Leaf types (described in the Result section) are mentioned for each *Oryza* species.

Genome	*Oryza* species	*IRGC* accession number	Leaf length (LL***, cm)	Leaf width (LW***, cm)	Category
KKLL	*O. coarctata*	104502	30.44±2.01 (lm)	0.68±0.08 (no)	Short-narrow
HHKK	*O. schlechteri*	82047	15.11±1.83 (q)	0.52±0.1 (o)	Short-narrow
HHJJ	*O longiglumis*	105148	32.22±2.86 (l)	1.53±0.09 (fg)	Short-wide
HHJJ	*O. ridleyi*	100821	36±3.61 (kl)	1.86±0.17 (d)	Short-wide
GG	*O meyeriana*	89241	16.33±1.5 (q)	1.88±0.23 (d)	Short-wide
GG	*O. granulata*	102118	17.67±2.12 (op)	1.92±0.17 (d)	Short-wide
FF	*O. brachyantha*	101232	18.78±1.09 (opq)	0.64±0.05 (no)	Short-narrow
EE	*O. australiensis*	100882	62.67±3.91 (cd)	1.42±0.14 (fgh)	Long-narrow
CCDD	*O grandiglumis*	106241	72.0±5.6 (ef)	2.61±0.58 (b)	Long-wide
CCDD	*O. latifolia*	105173	78.3±6.5 (gh)	2.7±0.1 (c)	Long-wide
CCDD	*O. alta*	105143	75.8±4.2 (bc)	3.28±0.71 (a)	Long-wide
CC	*O. rhizomatis*	105659	68.67±11.55 (nop)	2.94±0.2 (lm)	Long-wide
CC	*O. officinalis*	100896	58.2±19.4 (hij)	1.9±0.28 (de)	Long-wide
CC	*O. eichingeri*	101422	24.56±3.32 (no)	1.18±0.1 (ijk)	Short-wide
BBCC	*O. minuta*	101141	25.56±3.81 (mn)	1.31±0.18 (ghi)	Short-wide
BB	*O. punctata*	105690	62.22±6.1(ij)	1.4±0.11 (jkl)	Long-wide
AA	*O. glumaepatula*	106242	68.1±3.6 (ed)	1.49±0.27 (fg)	Long-wide
AA	*O. longistaminata*	110404	89.44±11.83 (a)	1.62±0.22 (ef)	Long-wide
AA	*O. rufipogon*	106424	74.5±1.6 (hij)	1.9±0.17 (hij)	Long-wide
AA	*O. meridionalis*	105301	53.22±11.12 (fg)	0.94±0.09 (lm)	Long-narrow
AA	*O. barthii*	106017	59.78±4.55 (ed)	0.98±0.07 (klm)	Long-narrow
AA	*O. nivara*	80723	47.56±7.06 (ghi)	0.83±0.1 (mn)	Long-narrow
AA	*O. glaberrima*	103544	60.7±4.2 (b)	1.2±0.1 (fg)	Long-narrow
AA	*O. sativa*	IR 64 - 21	41.00±8.51 (jk)	0.97±0.11 (klm)	Long-narrow

*** Represents significant difference among the species for the trait at $P<0.001$.

N = 9 Different letters suggest significant differences.
