# Supplementary material for: The Evolutionary Basis of Naturally Diverse Rice Leaves Anatomy
Source: PLoS One. 2016 Oct 28;11(10):e0164532. doi: 10.1371/journal.pone.0164532 (PMC5085062; doi:10.1371/journal.pone.0164532)
Supplement: S2 Table — (PDF) [file pone.0164532.s005.pdf]

S2 Table. Leaf thickness of *Oryza* species.

Leaf thickness (LT) is quantified by measuring vertical length of at least 3 transverse sections of 10 leaves per species (N = 30). LT values are represented as the average ± SD. LT varies significantly (*P*<0.0001) among the species.

Genome	*Oryza* species	*IRGC* accession number	Leaf thickness (LT***, µm)
KKLL	*O. coarctata*	104502	375.78±11 (a)
HHKK	*O. schlechteri*	82047	78.95±8.6 (ijk)
HHJJ	*O. longiglumis*	105148	75.34±3.8 (jkl)
HHJJ	*O. ridleyi*	100821	74.88±4.2 (kl)
GG	*O. meyeriana*	89241	69.3±5.1 (lm)
GG	*O. granulata*	102118	61.18±3.5 (n)
FF	*O. brachyantha*	101232	107.3±25.7 (d)
EE	*O. australiensis*	100882	116.86±15.4 (c)
CCDD	*O. grandiglumis*	106241	96.35±7.1 (e)
CCDD	*O. latifolia*	105173	84.88±4.4 (ghi)
CCDD	*O. alta*	105143	142.52±31.1 (b)
CC	*O. rhizomatis*	105659	81.99±3.5 (hij)
CC	*O. officinalis*	100896	86.49±9.8 (fgh)
CC	*O. eichingeri*	101422	78.75±11.2 (ijk)
BBCC	*O. minuta*	101141	109.69±10.9 (d)
BB	*O. punctata*	105690	53.76±4.2 (o)
AA	*O. glumaepatula*	106242	85.76±4.5 (ghi)
AA	*O. longistaminata*	110404	84.52±4.0 (hi)
AA	*O. rufipogon*	106424	91.76±9.2 (efg)
AA	*O. meridionalis*	105301	93.31±7.2 (ef)
AA	*O. barthii*	106017	72.68±6.3 (klm)
AA	*O. nivara*	80723	67.02±3.9 (mn)
AA	*O. glaberrima*	103544	65.68±7.7 (mn)
AA	*O. sativa* cv IR64	IR64-21	74.00±5.7 (lm)

*** Represents significant difference among the species for the trait at *P*<0.001. Different letters suggest significant differences.

N = 30
