# Supplementary material for: The Evolutionary Basis of Naturally Diverse Rice Leaves Anatomy
Source: PLoS One. 2016 Oct 28;11(10):e0164532. doi: 10.1371/journal.pone.0164532 (PMC5085062; doi:10.1371/journal.pone.0164532)
Supplement: S3 Table — (PDF) [file pone.0164532.s006.pdf]

S3 Table. Vein characters of *Oryza* species.

Veins (V) are counted at 1 mm lateral space at both the left and right side of the leaf, taking 10 leaves per species (N = 20). Vein related anatomical parameters are quantified by examining at least three leaf transverse sections per leaf. Values are presented as average ± SD. All the traits show significant (*P*<0.0001) difference in wild rice. Considering the consistent oval structure of veins in all the species, only the lateral diameter or the vein, 1.e., vein width is included for *ANOVA*.

Genome	*Oryza* species	*IRGC* accession number	Vein density (VD***, count)	Inter-veinal distance (IVD***, µm)	Inter-veinal mesophyll tissue length (TML = IVD X MCN, µm)	Vein width (VW*** , µm)	Vein height (VH, µm)
KKLL	*O. coarctata*	104502	5 ± 0.4 (efg)	226.2 ± 7.4 (cd)	85.08 ± 1.86	36.50 ± 4.3 (bc)	42 ± 3.45
HHKK	*O. schlechteri*	82047	6.4 ± 0.4 (ab)	153.43 ± 14.5 (l)	90.12 ± 1.8	22.32 ± 2.5 (l)	24.43 ± 3.62
HHJJ	*O. longiglumis*	105148	6.2 ± 0.5 (b)	147.92 ± 14.7 (m)	88.27§ ± 1.55	25.11 ± 4.04 (jk)	26.44 ± 3.17
HHJJ	*O. ridleyi*	100821	6.25 ± 0.7 (b)	162.97 ± 2.9 (jkl)	129.72 ± 2.86	22.51 ± 2.6 (kl)	29.5 ± 1.5
GG	*O. meyeriana*	89241	5.89 ± 1.09 (c)	199.9 ± 15.1 (fg)	126.21 ± 3.38	17.60 ± 2.2 (m)	22.29 ± 1.86
GG	*O. granulata*	102118	5.8 ± 0.5 (c)	159.74 ± 14.8 (kl)	67.17 ± 0.99	23.25 ± 1.7 (kl)	25 ± 1.5
FF	*O. brachyantha*	101232	6.67 ± 0.7 (a)	143.53 ± 25.8 (m)	88.22 ± 3.21	27.78 ± 4.9 (jkl)	25 ± 2.33
EE	*O. australiensis*	100882	5.15 ± 0.6 (ef)	166.4 ± 9.2 (ijk)	132.3 ± 2.58	31.95 ± 2.3 (cde)	37.5 ± 2.01
CCDD	*O. grandiglumis*	106241	5.5 ± 0.6 (d)	181.55 ± 11.0 9 (ij)	124.35 ± 2.89	29.24 ± 3.9 (hi)	31.75 ± 4.45
CCDD	*O. latifolia*	105173	4.9 ± 1.03 (fgh)	231.35 ± 10.24 (c)	173.68 ± 2.89	32.72 ± 2.9 (cdefg)	28.24 ± 2.83
CCDD	*O. alta*	105143	4.6 ± 0.4 (ij)	253 ± 39.1 (a)	202.65 ± 5.76	36.92 ± 4.5 (b)	47.72 ± 47.72
CC	*O. rhizomatis*	105659	5.6 ± 0.4 (cd)	218.6 ± 17.1 (de)	148.23 ± 1.9	27.88 ± 4.3 (gh)	29.55 ± 4.84
CC	*O. officinalis*	100896	4.9 ± 0.5 (d)	181.74 ± 8.9 (i)	108.16 ± 3.22	25.01 ± 2.8 (ij)	28.19 ± 3.01
CC	*O. eichingeri*	101422	5.2 ± 0.7 (e)	190.41 ± 11.3 (h)	123.77 ± 2.21	20.99 ± 3.1 (m)	24.75 ± 3.74
BBCC	*O. minuta*	101141	5.5 ± 0.4 (d)	172.82 ± 11.6 (ijk)	98.96 ± 0.84	31.85 ± 3.9 (cd)	37.5 ± 3.3
BB	*O. punctata*	105690	4.75 ± 0.7 (ghi)	174.59 ± 17.3 (i)	137.41 ± 1.35	17.16 ± 2.4 (m)	20.78 ± 2.74
AA	*O. glumaepatula*	106242	5.15 ± 0.4 (ef)	249.63 ± 17.0 7(b)	213.99 ± 2.02	40.06 ± 3.7 (a)	44.76 ± 6.23
AA	*O. longistaminata*	110404	3.85 ± 0.4 (k)	172.32 ± 33.3 (ij)	161.72 ± 4.08	32.68 ± 4.6 (fgh)	27.78 ± 4.47
AA	*O. rufipogon*	106424	4.4 ± 0.5 (j)	204.81 ± 18.5 (fg)	170.03 ± 5.22	31.83 ± 4.09 (defg)	40.31 ± 5.44
AA	*O. meridionalis*	105301	4.65 ± 0.9 (6)	205.66 ± 13.7 (fg)	137.06 ± 1.86	33.56 ± 5.3 (efgh)	39.38 ± 2.97
AA	*O. barthii*	106017	4.55 ± 0.7 (ij)	212.61 ± 19.6 (ef)	220.6 ± 3.1	28.95 ± 3.2 (cdef)	33.33 ± 3.55
AA	*O. nivara*	80723	5.5 ± 0.9 (d)	154.84 ± 12.4 (kl)	166.04 ± 0.58	25.51 ± 2.2 (jkl)	29.54 ± 2.79
AA	*O. glaberrima*	103544	5 ± 0.9 (ij)	229.91 ± 14.8 (cd)	231.34 ± 1.28	31.01 ± 3.8 (cd)	34.41 ± 4.3
AA	*O. sativa* cv IR64	IR64-21	5 ± 0.9 (ij)	196 ± 12.2 (gh)	157.54 ± 3.6	22.10 ± 3.6 (efgh)	29.5 ± 3.3

N = 30 (N = 20 for VD) *** Represents significant difference among the species for the trait at *P*<0.001. Different letters suggest significant differences.
