# Supplementary material for: The Evolutionary Basis of Naturally Diverse Rice Leaves Anatomy
Source: PLoS One. 2016 Oct 28;11(10):e0164532. doi: 10.1371/journal.pone.0164532 (PMC5085062; doi:10.1371/journal.pone.0164532)
Supplement: S4 Table — (PDF) [file pone.0164532.s007.pdf]

S4 Table. Mesophyll cell characters of *Oryza* species.

MC parameters are quantified examining at least 3 different sections per leaf and 10 leaves per species (N = 30). Values are presented as the average ± SD. All traits show significant (*P*<0.0001) difference among different wild rice.

Genome	*Oryza* species	*IRGC* accession number	Mesophyll cell number (MCN***, count)	Mesophyll cell length (MCL***, μm)	Mesophyll cell height (MCH***, μm)	Mesophyll cell width (MCW***, μm)	Mesophyll cell lobing (LB_{MC}***, ratio)
KKLL	*O. coarctata*	104502	4.00 ± 0.7 (j)	21.27 ± 2.6 (j)	14.49 ± 2.4 (cd)	7.74 ± 0.8 (cdef)	1.24 ± 0.1 (g)
HHKK	*O. schlechteri*	82047	6.00 ± 0.5 (e)	15.02 ± 3.3 (l)	10.51 ± 1.6 (ij)	9.78 ± 1.9 (c)	1.01 ± 0.03 (h)
HHJJ	*O. longiglumis*	105148	6.5 ± 0.6 (cd)	13.58 ± 2.3 (l)	10.07 ± 1.7 (j)	6.62 ± 1 (hijkl)	1.08 ± 0.1 (h)
HHJJ	*O. ridleyi*	100821	6.00 ± 0.7 (de)	21.62 ± 4.09 (hij)	13.72 ± 2.7 (ef)	7.70 ± 1.2 (cdef)	1.10 ± 0.1 (h)
GG	*O. meyeriana*	89241	7.42 ± 1.3 (a)	17.01 ± 2.4 (k)	15.07 ± 3.2 (d)	12.77 ± 3.1 (a)	1.07 ± 0.08 (h)
GG	*O. granulata*	102118	5.40 ± 0.5 (ghi)	12.44 ± 1.8 (l)	8.46 ± 5.09 (k)	10.9 ± 2.02 (b)	1 ± 0.08 (h)
FF	*O. brachyantha*	101232	6.20 ± 0.8 (de)	14.23 ± 3.8 (l)	11.02 ± 2.2 (hij)	7.24 ± 0.7 (efghij)	1.31 ± 0.1 (efg)
EE	*O. australiensis*	100882	5.4 ± 0.5 (hi)	24.50 ± 4.7 (efgh)	11.43 ± 1.9 (ij)	5.82 ± 0.6 (klmn)	1.54 ± 0.1 (bcd)
CCDD	*O. grandiglumis*	106241	4.53 ± 0.6 (j)	27.45 ± 4.5 (d)	15.27 ± 1.8 (cd)	6.63 ± 0.9 (jklm)	1.46 ± 0.1 (bcde)
CCDD	*O. latifolia*	105173	5.77 ± 0.6 (ef)	30.10 ± 4.3 (cd)	17.36 ± 1.9 (bc)	7.18 ± 0.6 (fghij)	1.36 ± 0.02 (efg)
CCDD	*O. alta*	105143	5.48 ± 0.7 (i)	36.98 ± 7.38 (a)	19.34 ± 3.6 (a)	8.44 ± 0.7 (cd)	1.49 ± 0.1 (cdef)
CC	*O. rhizomatis*	105659	6.53 ± 0.5 (c)	22.70 ± 3.7 (ij)	12.36 ± 1.5 (ghi)	7.37 ± 1.2 (defg)	1.43 ± 0.1 (cdef)
CC	*O. officinalis*	100896	5.31 ± 0.7 (i)	20.37 ± 4.07 (j)	12.70 ± 1.9 (efg)	4.9 ± 0.7 (mn)	1.39 ± 0.1 (cdef)
CC	*O. eichingeri*	101422	5.73 ± 0.5 (fgh)	21.60 ± 4.1 (j)	13.21 ± 1.3 (d)	7.33 ± 0.5 (efghi)	1.39 ± 0.09 (defg)
BBCC	*O. minuta*	101141	4.00 ± 0.7 (j)	24.74 ± 1.2 (e)	12.63 ± 2.07 (fghi)	10.19 ± 0.9 (b)	1.54 ± 0.2 (b)
BB	*O. punctata*	105690	6.11 ± 0.3 (e)	22.49 ± 4.08 (fghij)	12.07 ± 1.6 (fghi)	7.51 ± 0.8 (efghi)	1.33 ± 0.09 (fg)
AA	*O. glumaepatula*	106242	6.85 ± 0.4 (ab)	31.24 ± 4.7 (b)	15.89 ± 1.6 (b)	5.62 ± 0.8 (lmn)	1.51 ± 0.1 (bc)
AA	*O. longistaminata*	110404	6.50 ± 0.7 (cd)	24.88 ± 5.1 (ef)	13.12 ± 2.6 (efghi)	7.97 ± 0.7 (cdef)	1.48 ± 0.1 (cdef)
AA	*O. rufipogon*	106424	7.37 ± 0.7 (a)	23.07 ± 6.9 (ghij)	13.22 ± 1.6 (e)	6.6 ± 0.6 (defgh)	1.57 ± 0.1 (bc)
AA	*O. meridionalis*	105301	5.88 ± 0.5 (efg)	23.31 ± 3.6 (ef)	13.27 ± 1.6 (ef)	6.95 ± 0.6 (ijkl)	1.72 ± 0.1 (a)
AA	*O. barthii*	106017	7.16 ± 0.8 (ab)	30.81 ± 3.8 (bc)	12.63 ± 1.7 (efgh)	6.24 ± 1.2 (ijkl)	1.53 ± 0.1 (bc)
AA	*O. nivara*	80723	7.00 ± 0.2 (bc)	23.72 ± 2.9 (efghi)	12.51 ± 1.9 (efgh)	4.99 ± 0.8 (n)	1.60 ± 0.2 (b)
AA	*O. glaberrima*	103544	7.14 ± 0.2 (ab)	32.40 ± 6.4 (b)	12.01 ± 1.7 (fghi)	7.06 ± 0.7 (ghijk)	1.52 ± 0.1 (cdef)
AA	*O. sativa* cv IR64	IR64-21	6.60 ± 0.8 (bc)	23.87 ± 4.4 (efg)	12.54 ± 1.9 (efg)	7.80 ± 1.02 (cde)	1.56 ± 0.1 (bc)

***Represents significant difference among the species for the trait at *P*<0.001. Different letters suggest significant differences.

N = 30
