# Supplementary material for: The Evolutionary Basis of Naturally Diverse Rice Leaves Anatomy
Source: PLoS One. 2016 Oct 28;11(10):e0164532. doi: 10.1371/journal.pone.0164532 (PMC5085062; doi:10.1371/journal.pone.0164532)
Supplement: S5 Table — (PDF) [file pone.0164532.s008.pdf]

**S5 Table. Bundle sheath cell characters of *Oryza* species.**

BSC parameters are measured examining at least 3 different sections from 10 different leaves per species (N = 30). All traits except the BSC height showed significant (*P*<0.0001) difference in wild rice.

| Genome | *Oryza* species | *IRGC* accession number | Bundle sheath cell number (BSCN***, count) | Bundle sheath cell width (BSCW***, μm) | Bundle sheath cell height (BSCH, μm) | Bundle sheath cell length (BSCL***, μm) |
|---|---|---|---|---|---|---|
| KKLL | *O. coarctata* | 104502 | 7.91 ± 0.9 (fg) | 45.05 ± 8.6 (a) | 38.32 ± 12.01 | 29.13 ± 2.5 (j) |
| HHKK | *O. schlechteri* | 82047 | 6.80 ± 0.3 (h) | 13.19 ± 3.67 (c) | 16.92 ± 3.03 | 40.13 ± 8.47 (efgh) |
| HHJJ | *O. longiglumis* | 105148 | 6.64 ± 0.4 (h) | 13.51 ± 2.6 (de) | 15.02 ± 2.86 | 69.14 ± 11.2 (bc) |
| HHJJ | *O. ridleyi* | 100821 | 7.00 ± 1.1 (h) | 10.32 ± 1.6 (hijk) | 12.77 ± 1.26 | 46.89 ± 9.03 (fghi) |
| GG | *O. meyeriana* | 89241 | 7.25 ± 0.9 (gh) | 10.96 ± 1.8 (ijk) | 12.18 ± 2.43 | 67.35 ± 8.7 (bc) |
| GG | *O. granulata* | 102118 | 7.10 ± 0.8 (h) | 10.00 ± 3.3 (fghij) | 11.61 ± 2.13 | 57.00 ± 5.9 (cd) |
| FF | *O. brachyantha* | 101232 | 8.00 ± 0.9 (ef) | 12.01 ± 2.5 (def) | 13.75 ± 2.81 | 46.74 ± 4.9 (efgh) |
| EE | *O. australiensis* | 100882 | 8.14 ± 0.3 (def) | 12.00 ± 1.7 (efghi) | 14.84 ± 2.38 | 61.02 ± 14.9 (cde) |
| CCDD | *O. grandiglumis* | 106241 | 8.80 ± 0.3 (cd) | 13.00 ± 2.04 (efghi) | 13.28 ± 2.25 | 59.73 ± 13.9 (cdef) |
| CCDD | *O. latifolia* | 105173 | 9.50 ± 1 (c) | 13.86 ± 2.28 (cde) | 15.24 ± 2.31 | 67.78 ± 11.2 (bcd) |
| CCDD | *O. alta* | 105143 | 11.50 ± 0.9 (a) | 20.18 ± 3.7 (b) | 20.93 ± 2.97 | 66.43 ± 15.2 (cdef) |
| CC | *O. rhizomatis* | 105659 | 8.33 ± 0.7 (def) | 11.54 ± 2.5 (efghi) | 12.20 ± 1.98 | 47.63 ± 6.5 (fgh) |
| CC | *O. officinalis* | 100896 | 8.14 ± 0.6 (def) | 11.93 ± 1.5 (efgh) | 13.91 ± 3.56 | 62.57 ± 9.8 (bcd) |
| CC | *O. eichingeri* | 101422 | 8.20 ± 0.3 (def) | 11.56 ± 2.3 (cd) | 12.86 ± 2.63 | 83.77 ± 10.1 (a) |
| BBCC | *O. minuta* | 101141 | 8.16 ± 1.1 (d) | 15.81 ± 0.92 (c) | 15.84 ± 3.83 | 59.00 ± 8.7 (cdef) |
| BB | *O. punctata* | 105690 | 7.60 ± 1.1 (de) | 9.65 ± 2.8 (ghij) | 10.26 ± 1.57 | 72.47 ± 10.09 (ab) |
| AA | *O. glumaepatula* | 106242 | 11.60 ± 0.5 (a) | 12.40 ± 1.8 (efg) | 14.31 ± 2.13 | 41.00 ± 12.5 (hi) |
| AA | *O. longistaminata* | 110404 | 11.25 ± 1.1 (a) | 10.93 ± 2.2 (ijk) | 12.08 ± 2.83 | 57.16 ± 6.2 (ghi) |
| AA | *O. rufipogon* | 106424 | 12.50 ± 0.7 (a) | 10.18 ± 2.4 (ijk) | 10.64 ± 2.02 | 60.65 ± 15.04 (cdef) |
| AA | *O. meridionalis* | 105301 | 11.60 ± 0.9 (a) | 10.49 ± 2.1 (hijk) | 11.93 ± 2.28 | 65.33 ± 10.7 (bcd) |
| AA | *O. barthii* | 106017 | 10.20 ± 0.5 (b) | 8.95 ± 1.9 (jk) | 10.42 ± 2.4 | 53.31 ± 15.8 (defg) |
| AA | *O. nivara* | 80723 | 11.50 ± 0.7 (a) | 8.78 ± 1.4 (k) | 10.35 ± 1.5 | 49.06 ± 10.9 (ghi) |
| AA | *O. glaberrima* | 103544 | 11.18 ± 1.3 (a) | 9.36 ± 1.9 (fghij) | 10.90 ± 1.8 | 45.12 ± 12.3 (ij) |
| AA | *O. sativa* cv IR64 | IR64-21 | 12.50 ± 0.5 (a) | 9.57 ± 2.01 (jk) | 11.24 ± 2.01 | 53.55 ± 11.01 (ghi) |

*** Represents significant difference among the species for the trait at *P*<0.001. Different letters suggest significant differences.

N = 30
