# Supplementary material for: The Evolutionary Basis of Naturally Diverse Rice Leaves Anatomy
Source: PLoS One. 2016 Oct 28;11(10):e0164532. doi: 10.1371/journal.pone.0164532 (PMC5085062; doi:10.1371/journal.pone.0164532)
Supplement: S6 Table — (PDF) [file pone.0164532.s009.pdf]

S6 Table. Detailed anatomical characters of three high yielding *Oryza sativa* cultivars IR64, IR24 and IR31917.

Cultivars	Leaf thickness, LT)	Inter-veinal distance (IVD, µm)	Inter-veinal total mesophyll length (TML, µm)	Mesophyll cell number (MCN, count)	Mesophyll cell length (MCL, µm)	Mesophyll cell height (MCH, µm)	Mesophyll cell width (MCW, µm)	Mesophyll cell lobing (LB_{MC}, ratio)
O. sativa cv IR64	74 ± 5.7	196 ± 12.2	157.54 ± 3.6	6.6 ± 0.8	23.87 ± 4.4	12.54 ± 1.9	7.8 ± 1.02	1.56 ± 0.1
O. sativa cv IR24	87.63 ± 7.57	213.12 ± 21.22	169.3 ± 2.42	6.6 ± 0.58	25.65 ± 4.19	14.93 ± 1.07	7.88 ± 1.13	1.5 ± 0.1
O. sativa cv IR31917	77.86 ± 6.51	221.1 ± 12.06	190.64 ± 1.77	7 ± 0.4	27.23 ± 4.41	12.43 ± 1.3	6.23 ± 0.76	1.59 ± 0.09
N = 30, 20 for VD								

S6 Table continued.

Cultivars	Bundle sheath cell number (BSCN, count)	Bundle sheath cell width (BSCW, µm)	Bundle sheath cell height (BSCH, µm)	Bundle sheath cell length (BSCL, µm)	Vein density (VD, count)	Vein width (VW, µm)	Vein height (VH, µm)
O. sativa cv IR64	12.5 ± 0.5	9.57 ± 2.01	11.24 ± 2.01	53.55 ± 11.01	5 ± 0.9	22.1 ± 3.6	29.5 ± 3.3
O. sativa cv IR24	12.4 ± 0.5	12.03 ± 1.42	12.66 ± 1.77	68.4 ± 16.97	5 ± 0.5	33.76 ± 5.98	33.5 ± 2.3
O. sativa cv IR31917	12.0 ± 0.5	12.92 ± 1.56	13.88 ± 1.39	52.7 ± 11.4	5 ± 0.4	27.89 ± 1.69	29.5 ± 1.25
N = 30, 20 for VD							

All the anatomical parameters are as described in Material and Methods. Traits are quantified from the data collected from 10 leaves per accession and 3 sections (where required) per leaf. Values are the average ± SD.
