# Supplementary material for: The Evolutionary Basis of Naturally Diverse Rice Leaves Anatomy
Source: PLoS One. 2016 Oct 28;11(10):e0164532. doi: 10.1371/journal.pone.0164532 (PMC5085062; doi:10.1371/journal.pone.0164532)
Supplement: S7 Table — (PDF) [file pone.0164532.s010.pdf]

S7 Table. Detailed anatomical characters of distant wild rice species.

Species	*IRGC* Accession number	Leaf thickness (LT, μm)	Inter-veinal distance (IVD, μm)	Inter-veinal total mesophyll length (TML, μm)	Mesophyll cell number (MCN, count)	Mesophyll cell length (MCL, μm)	Mesophyll cell height (MCH, μm)
Hygroryza aristata	105457	91.98 ± 7.66	296.24 ± 7.6	142.01 ± 15.31	7.33 ± 1.15	20.37 ± 4.78	11.15 ± 2.07
Luziola leiocarpa	82043	66.08 ± 6.5	141.85 ± 5.92	90.21 ± 7.78	5.38 ± 0.52	17.24 ± 3.97	10.06 ± 1.26
Rhynchoryza subulata	100913	146.83 ± 11.97	169.56 ± 8.56	94.82 ± 13.37	5.2 ± 0.45	16.05 ± 3.42	10.6 ± 1.5
Zizanopsis villanensis	85425	189.21 ± 4.29	234.98 ± 7.02	119.57 ± 36.89	5.4 ± 0.89	21.59 ± 3.01	15.35 ± 2.65
Chikusichloa aquatica	106186	107.7 0± 13.9	241.78 ± 33.22	83.14 ± 4.49	4.5 ± 0.58	24.43 ± 5.74	8.91 ± 0.68
Leersia tisseranti	101384	75.31 ± 6.93	161.76 ± 15.2	109.79 ± 14.69	6.38 ± 0.52	17.23 ± 3.26	11.45 ± 1.83

N = 30

S7 Table continued.

Species	IRGC Accession number	Mesophyll cell lobing (LB_{MC}, μm)	Bundle sheath cell number (BSCN, count)	Bundle sheath cell width (BSCW, μm)	Bundle sheath cell height (BSCH, μm)	Vein width (VW, μm)	Vein height (VH, μm)
Hygroryza aristata	105457	1 ± 0.1	8.14 ± 0.38	14.66 ± 3.57	16.86 ± 3.71	26.33 ± 4.61	31.42 ± 2.65
Luziola leiocarpa	82043	1.17 ± 0.13	8.13 ± 0.35	7.90 ± 1.28	9.37 ± 1.62	23.31 ± 2.63	25.21 ± 1.65
Rhynchoryza subulata	100913	1.25 ± 0.09	12.2 ± 1.3	10.91 ± 1.91	11.78 ± 2.61	34.38 ± 5.98	58.44 ± 12.58
Zizanopsis villanensis	85425	1.48 ± 0.09	12 ± 1.41	21.78 ± 2.89	21.27 ± 2.5	55.19 ± 9.17	85.76 ± 9.5
Chikusichloa aquatica	106186	1.12 ± 0.06	8.83 ± 1.17	16.37 ± 3.84	14.17 ± 2.36	28.57 ± 3.01	37.26 ± 5.43
Leersia tisseranti	101384	1.38 ± 0.13	9.29 ± 0.49	8.64 ± 1.85	10.05 ± 1.37	20.49 ± 2.55	24.86 ± 2.47

N = 30

All the anatomical parameters are as described in Material and Methods. Traits are quantified from 3 sections per leaves and 10 leaves per species. Values are presented as the average ± SD.
