# Supplementary material for: The Evolutionary Basis of Naturally Diverse Rice Leaves Anatomy
Source: PLoS One. 2016 Oct 28;11(10):e0164532. doi: 10.1371/journal.pone.0164532 (PMC5085062; doi:10.1371/journal.pone.0164532)
Supplement: S8 Table — (PDF) [file pone.0164532.s011.pdf]

S8 Table. ***NCBI*** **accessions of the genes used in constructing the rice phylogenetic tree.**

<table>
<thead>
<tr><th>Serial number</th><th>NCBI accession numbers of genes used in constructing Oryza phylogenetic tree</th></tr>
</thead>
<tbody>
<tr><td>1</td><td>gi|61099007|gb|AY792585.1| Rhynchoryza subulata alcohol dehydrogenase II (Adh2) gene, exons 3 through 7 and partial cds</td></tr>
<tr><td>2</td><td>>gi|7188539|gb|AF148628.1| Porteresia coarctata alcohol dehydrogenase II (Adh2) gene, exons 3 through 7, partial cds</td></tr>
<tr><td>3</td><td>>gi|7188541|gb|AF148629.1| Oryza schlechteri alcohol dehydrogenase II (Adh2) gene, exons 3 through 7, partial cds</td></tr>
<tr><td>4</td><td>>gi|7188533|gb|AF148625.1| Oryza longiglumis alcohol dehydrogenase II (Adh2) gene, exons 3 through 7, partial cds</td></tr>
<tr><td>5</td><td>>gi|7188531|gb|AF148624.1| Oryza ridleyi alcohol dehydrogenase II (Adh2) gene, exons 3 through 7, partial cds</td></tr>
<tr><td>6</td><td>>gi|7188543|gb|AF148630.1| Oryza meyeriana alcohol dehydrogenase II (Adh2) gene, exons 3 through 7, partial cds</td></tr>
<tr><td>7</td><td>>gi|7188545|gb|AF148631.1| Oryza granulata alcohol dehydrogenase II (Adh2) gene, exons 3 through 7, partial cds</td></tr>
<tr><td>8</td><td>>gi|7188547|gb|AF148632.1| Oryza brachyantha alcohol dehydrogenase II (Adh2) gene, exons 3 through 7, partial cds</td></tr>
<tr><td>9</td><td>>gi|7188529|gb|AF148623.1| Oryza australiensis alcohol dehydrogenase II (Adh2) gene, exons 3 through 7, partial cds</td></tr>
<tr><td>10</td><td>>gi|7188527|gb|AF148622.1| Oryza grandiglumis alcohol dehydrogenase II (Adh2) gene, exons 3 through 7, partial cds</td></tr>
<tr><td>11</td><td>>gi|7188525|gb|AF148621.1| Oryza latifolia alcohol dehydrogenase II (Adh2) gene, exons 3 through 7, partial cds</td></tr>
<tr><td>12</td><td>>gi|7188518|gb|AF148617.1| Oryza alta alcohol dehydrogenase II (Adh2) gene, exons 3 through 7, partial cds</td></tr>
<tr><td>13</td><td>>gi|7188512|gb|AF148614.1| Oryza rhizomatis alcohol dehydrogenase II (Adh2) gene, exons 3 through 7, partial cds</td></tr>
<tr><td>14</td><td>>gi|7188510|gb|AF148613.1| Oryza officinalis alcohol dehydrogenase II (Adh2) gene, exons 3 through 7, partial cds</td></tr>
<tr><td>15</td><td>>gi|7188514|gb|AF148615.1| Oryza eichingeri alcohol dehydrogenase II (Adh2) gene, exons 3 through 7, partial cds</td></tr>
<tr><td>16</td><td>>gi|7188516|gb|AF148616.1| Oryza minuta alcohol dehydrogenase II (Adh2) gene, exons 3 through 7, partial cds</td></tr>
<tr><td>17</td><td>>gi|7188506|gb|AF148611.1| Oryza punctata alcohol dehydrogenase II (Adh2) gene, exons 3 through 7, partial cds</td></tr>
<tr><td>18</td><td>>gi|7188494|gb|AF148605.1| Oryza glumaepatula alcohol dehydrogenase II (Adh2) gene, exons 3 through 7, partial cds</td></tr>
<tr><td>19</td><td>>gi|7188498|gb|AF148607.1| Oryza longistaminata alcohol dehydrogenase II (Adh2) gene, exons 3 through 7, partial cds</td></tr>
<tr><td>20</td><td>>gi|7188490|gb|AF148603.1| Oryza rufipogon alcohol dehydrogenase II (Adh2) gene, exons 3 through 7, partial cds</td></tr>
<tr><td>21</td><td>>gi|7188502|gb|AF148609.1| Oryza meridionalis alcohol dehydrogenase II (Adh2)</td></tr>
</tbody>
</table>

gene, exons 3 through 7, partial cds
gi|7188500|gb|AF148608.1| Oryza barthii alcohol dehydrogenase II (Adh2) gene,
22 exons 3 through 7, partial cds
>gi|7188492|gb|AF148604.1| Oryza nivara alcohol dehydrogenase II (Adh2) gene,
23 exons 3 through 7, partial cds
>gi|7188496|gb|AF148606.1| Oryza glaberrima alcohol dehydrogenase II (Adh2)
24 gene, exons 3 through 7, partial cds
>gi|7188488|gb|AF148602.1| Oryza sativa alcohol dehydrogenase II (Adh2) gene,
25 exons 3 through 7, partial cds
gi|7158358|gb|AF148599.1| Rhynchoryza subulata alcohol dehydrogenase I (Adh1)
26 gene, exons 3 through 7, partial cds
gi|7158346|gb|AF148593.1| Porteresia coarctata alcohol dehydrogenase I (Adh1)
27 gene, exons 3 through 7, partial cds
gi|7158344|gb|AF148592.1| Oryza schlechteri alcohol dehydrogenase I (Adh1)
28 gene, exons 3 through 7, partial cds
gi|7158340|gb|AF148590.1| Oryza ridleyi H type alcohol dehydrogenase I (Adh1)
29 gene, exons 3 through 7, partial cds
gi|7158342|gb|AF148591.1| Oryza longiglumis H type alcohol dehydrogenase I
30 (Adh1) gene, exons 3 through 7, partial cds
gi|7158352|gb|AF148596.1| Oryza meyeriana alcohol dehydrogenase I (Adh1)
31 gene, exons 3 through 7, partial cds
gi|7158354|gb|AF148597.1| Oryza granulata alcohol dehydrogenase I (Adh1) gene,
32 exons 3 through 7, partial cds
gi|7158356|gb|AF148598.1| Oryza brachyantha alcohol dehydrogenase I (Adh1)
33 gene, exons 3 through 7, partial cds
gi|7158338|gb|AF148589.1| Oryza australiensis alcohol dehydrogenase I (Adh1)
34 gene, exons 3 through 7, partial cds
gi|7158336|gb|AF148588.1| Oryza grandiglumis D type alcohol dehydrogenase I
35 (Adh1) gene, exons 3 through 7, partial cds
gi|7158334|gb|AF148587.1| Oryza latifolia D type alcohol dehydrogenase I (Adh1)
36 gene, exons 3 through 7, partial cds
gi|7158326|gb|AF148583.1| Oryza alta C type alcohol dehydrogenase I (Adh1)
37 gene, exons 3 through 7, partial cds
gi|7158320|gb|AF148580.1| Oryza rhizomatis alcohol dehydrogenase I (Adh1)
38 gene, exons 3 through 7, partial cds
gi|7158312|gb|AF148576.1| Oryza eichingeri alcohol dehydrogenase I (Adh1) gene,
39 exons 3 through 7, partial cds
gi|7158318|gb|AF148579.1| Oryza officinalis alcohol dehydrogenase I (Adh1) gene,
40 exons 3 through 7, partial cds
gi|7158324|gb|AF148582.1| Oryza minuta C type alcohol dehydrogenase I (Adh1)
41 gene, exons 3 through 7, partial cds
gi|7158314|gb|AF148577.1| Oryza punctata alcohol dehydrogenase I (Adh1) gene,
42 exons 3 through 7, partial cds
gi|7158298|gb|AF148569.1| Oryza rufipogon alcohol dehydrogenase I (Adh1) gene,
43 exons 3 through 7, partial cds
44 gi|7158300|gb|AF148570.1| Oryza nivara alcohol dehydrogenase I (Adh1) gene,

exons 3 through 7, partial cds
gi|7158310|gb|AF148575.1| Oryza meridionalis alcohol dehydrogenase I (Adh1)
45 gene, exons 3 through 7, partial cds
gi|7158306|gb|AF148573.1| Oryza longistaminata alcohol dehydrogenase I (Adh1)
46 gene, exons 3 through 7, partial cds
gi|7158302|gb|AF148571.1| Oryza glumaepatula alcohol dehydrogenase I (Adh1)
47 gene, exons 3 through 7, partial cds
gi|7158308|gb|AF148574.1| Oryza barthii alcohol dehydrogenase I (Adh1) gene,
48 exons 3 through 7, partial cds
gi|7158304|gb|AF148572.1| Oryza glaberrima alcohol dehydrogenase I (Adh1)
49 gene, exons 3 through 7, partial cds
gi|7158296|gb|AF148568.1| Oryza sativa subsp. japonica alcohol dehydrogenase I
50 (Adh1) gene, exons 3 through 7, partial cds
