# Supplementary material for: The Evolutionary Basis of Naturally Diverse Rice Leaves Anatomy
Source: PLoS One. 2016 Oct 28;11(10):e0164532. doi: 10.1371/journal.pone.0164532 (PMC5085062; doi:10.1371/journal.pone.0164532)
Supplement: S9 Table — (PDF) [file pone.0164532.s012.pdf]

S9 Table : Phylogenetic signal in the leaf traits.

Investigation of dependency of trait values on phylogeny using two methods: 1) whether phylogenetic distances between *Oryza* species correlate to corresponding differences in values of traits and 2) measurement of phylogenetic signal using K method. The spearman correlation (rho) was computed using custom perl scripts. To obtain p-value for these correlations, the difference in trait values was randomized 10,000 times to obtain number of times the correlation from randomization exceeds original correlation. To test the reliability of these correlations, p-value was obtained by randomisation, which showed that most of them are significant, though the correlations are poor to give any strong phylogenetic signal. In the second approach, phylogenetic signal too showed a similar pattern where none has a value of K >=1.

**Trait**	**Spearman Correlation** (between phylogenetic distance and difference in values of a trait)	**P(x>=rho)** (randomisation)	**Phylogenetic signal (K)**
Leaf length	0.41	0	0.11
Leaf width	0.19	0.124	0.05
Leaf thickness	0.32	0	0.08
Vein density	0.44	0	0.13
Inter-veinal distance	0.09	0.181	0.03
Vein width	0.11	0.143	0.13
Vein height	0.09	0.172	0.06
Mesophyll cell number	0.18	0.337	0.11
Mesophyll cell length	0.53	0	0.22
Mesophyll cell height	0.45	0	0.07
Mesophyll cell width	0.27	0.003	0.11
Mesophyll cell lobing	0.64	0	0.95
Bundle sheath cell number	0.42	0	0.5
Bundle sheath cell length	0.05	0.302	0.12
Bundle sheath cell height	0.06	0.259	0.11
Bundle sheath cell width	-0.02	0.408	0.06
